# ROS-producing immature neutrophils in giant cell arteritis are linked to vascular pathologies

**DOI:** 10.1172/jci.insight.139163

**Published:** 2020-10-15

**Authors:** Lihui Wang, Zhichao Ai, Tariq Khoyratty, Kristina Zec, Hayley L. Eames, Erinke van Grinsven, Alison Hudak, Susan Morris, David Ahern, Claudia Monaco, Evgeniy B. Eruslanov, Raashid Luqmani, Irina A. Udalova

**Affiliations:** 1 Kennedy Institute of Rheumatology and; 2 Botnar Research Centre, Department of Orthopaedics, Rheumatology and Musculoskeletal Science, University of Oxford, Headington, Oxford, United Kingdom.; 3 Division of Thoracic Surgery, Department of Surgery, Perelman School of Medicine at the University of Pennsylvania, Philadelphia, Pennsylvania, USA.

**Keywords:** Vascular Biology, Autoimmune diseases, Neutrophils, Vasculitis

## Abstract

Giant cell arteritis (GCA) is a common form of primary systemic vasculitis in adults, with no reliable indicators of prognosis or treatment responses. We used single cell technologies to comprehensively map immune cell populations in the blood of patients with GCA and identified the CD66b^+^CD15^+^CD10^lo/–^CD64^–^ band neutrophils and CD66b^hi^CD15^+^CD10^lo/–^CD64^+/bright^ myelocytes/metamyelocytes to be unequivocally associated with both the clinical phenotype and response to treatment. Immature neutrophils were resistant to apoptosis, remained in the vasculature for a prolonged period of time, interacted with platelets, and extravasated into the tissue surrounding the temporal arteries of patients with GCA. We discovered that immature neutrophils generated high levels of extracellular reactive oxygen species, leading to enhanced protein oxidation and permeability of endothelial barrier in an in vitro coculture system. The same populations were also detected in other systemic vasculitides. These findings link functions of immature neutrophils to disease pathogenesis, establishing a clinical cellular signature of GCA and suggesting different therapeutic approaches in systemic vascular inflammation.

## Abstract 2

Bona fide immature neutrophil subsets produce unchecked extracellular ROS that contributes to vascular pathologies.

## Graphical abstract

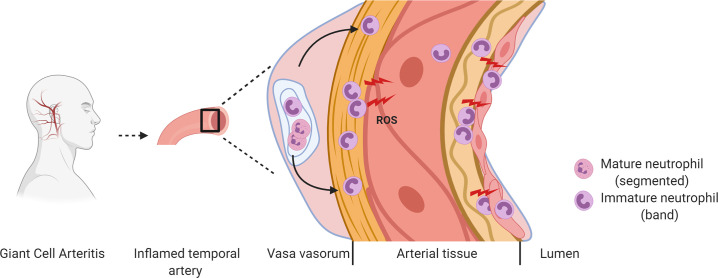

## Introduction

The systemic vasculitides are a group of autoimmune disorders characterized by inflammation of blood vessels, resulting in end organ damage and — in many untreated cases — death. The severest forms of vasculitis are typically associated with antineutrophil cytoplasm antibodies (ANCAs) and, thus, collectively termed ANCA-associated vasculitides (AAV) (1), such as granulomatosis with polyangiitis (GPA, previously referred to as Wegener’s granulomatosis). In other forms of vasculitis such as giant cell arteritis (GCA), where autoantibodies are absent, the disease can lead to early and permanent visual loss, as well as aortic aneurysms in the long-term if inadequately recognized without timely intervention. The disease burden of GCA, which is as common as rheumatoid arthritis (RA), in the growing global aging population is considerable and has been estimated up to $84 billion in the USA alone by 2050 (2).

In AAV, ANCAs are a reliable biomarker in diagnosis, but their role in predicting response to therapy, disease activity, or relapses remains uncertain. ANCAs are known to play a central role in its pathogenesis of AAV because the autoantibodies induce excessive activation of neutrophils, causing damage to small vessels (3). In contrast, the cellular and molecular mechanisms of GCA remain largely elusive. Progress has been slow in developing reliable techniques to monitor disease activity, progression, and treatment response (4, 5). The GCA diagnosis is based on histological examination or imaging to demonstrate inflammation of the affected vessels (6), as well as the identification of macrophages and T cells and the production of inflammatory and growth signals in the lesions (7, 8). Proinflammatory cytokines such as IL-6 and its soluble receptors, pattern recognition receptor pentraxin-3 and vascular endothelial growth factor (VEGF), have been suggested as biomarkers in GCA (9, 10). However, they fail to differentiate between disease activity and concomitant infection or other causes of acute inflammation. Consequently, the standard care of GCA is largely limited to nonspecific glucocorticoid therapy; however, toxicity from high-dose glucocorticoids often results in serious comorbidity, including secondary infection, type II diabetes mellitus, and hypertension, posing a significant challenge to the patient’s quality of life. Establishing effective tools and systems to identify cells and molecules that may shape the disease would provide a framework to selectively target control mechanisms, which govern their functions. Recently, a comprehensive leukocyte profiling in patients with GCA who were studied from disease onset to remission by van Sleen et al. has clearly demonstrated an elevated and persistent neutrophil presence during the entire disease course, pointing to the potential application of neutrophils as the cellular marker in the prognosis of GCA (11).

Previous studies, including those from our own group, have already suggested a role for neutrophils in autoimmunity (12–14) and highlight context-specific diversity in neutrophil identity, plasticity, and function (15). Neutrophil heterogeneity is now a recognized phenomenon associated closely with several diseases. However, evidence of unequivocal characterization of bona fide neutrophil subsets, particularly in the context of pathological conditions, has not yet been reported, to our knowledge (14–16).

In steady state, neutrophils are essential components of innate immunity. They exert their conventional antimicrobial activity through well-established mechanisms including release of cytotoxic products, reactive oxygen species (ROS), neutrophil extracellular traps (NETs), and pore-forming molecules (17).These activities cause tissue damage, if poorly controlled. Recent progress has revealed that neutrophils are also important components of myeloid regulatory cells, playing essential roles in immune homeostasis — not only systemically via the circulation (18), but also locally in naive tissues (19). In health, neutrophils mature in BM and are released as mature granulocytes. However, in pathological conditions, this process can be altered under the influence of cytokines and growth factors produced locally or systemically. This leads to the release of immature neutrophils into the circulation and tissue, detectable in the blood of patients with autoimmune diseases and cancer (20).

Currently, neutrophil populations are identified and classified based on their phenotypical and physical changes, and their associated immune functions dependent on experimental or clinical settings. In GCA, Nadkarni and colleagues have made seminal discoveries on the dynamic and functions of neutrophils in response to treatment with glucocorticoids and identified a neutrophil subset in the circulation, which suppressed T cell proliferation (21). In GPA, low-density neutrophils (LDNs) were reported to significantly increase in number and were speculated to play pathogenic roles via spontaneous NET formation (22). LDNs are an heterogeneous group of mature and immature neutrophils that were initially discovered to be enriched with mononuclear cells from PBMC during Ficoll density gradient centrifugation resulting from their reduced cellular density (23). LDNs have been reported to increase significantly in other chronic inflammatory conditions, such as systemic lupus erythematosus (SLE) (23–25) and RA (23, 26). In SLE, LDNs are considered to be highly proinflammatory and have been reported to undergo spontaneous NET release, which is speculated to be one of the immunopathogenic mechanisms of the disease (27). However, LDNs derived from patients with RA were reported to be less proinflammatory and largely functionally immature and defective (26). Apart from the established role of ANCAs in excessive and systemic activation of neutrophils, the precise pathophysiological roles and functions of neutrophils and LDNs in the systemic vasculitides are largely unknown.

In this study, we identified immature neutrophils as the cells that were characteristic of the patients with GCA, as well as GPA; remained in the vasculature for a prolonged time; interacted with platelets; extravasated into the tissue surrounding the temporal arteries; and generated high levels of extracellular ROS, affecting vascular barrier. These findings contribute to better understanding of GCA pathophysiology and lead to previously unrecognized therapeutic approaches to systemic vascular inflammation.

## Results

### Cell surface expression of CD66b, CD15, CD16, CD10, and CD64 distinguish vasculitis-associated LDN populations.

**Figure 1:**
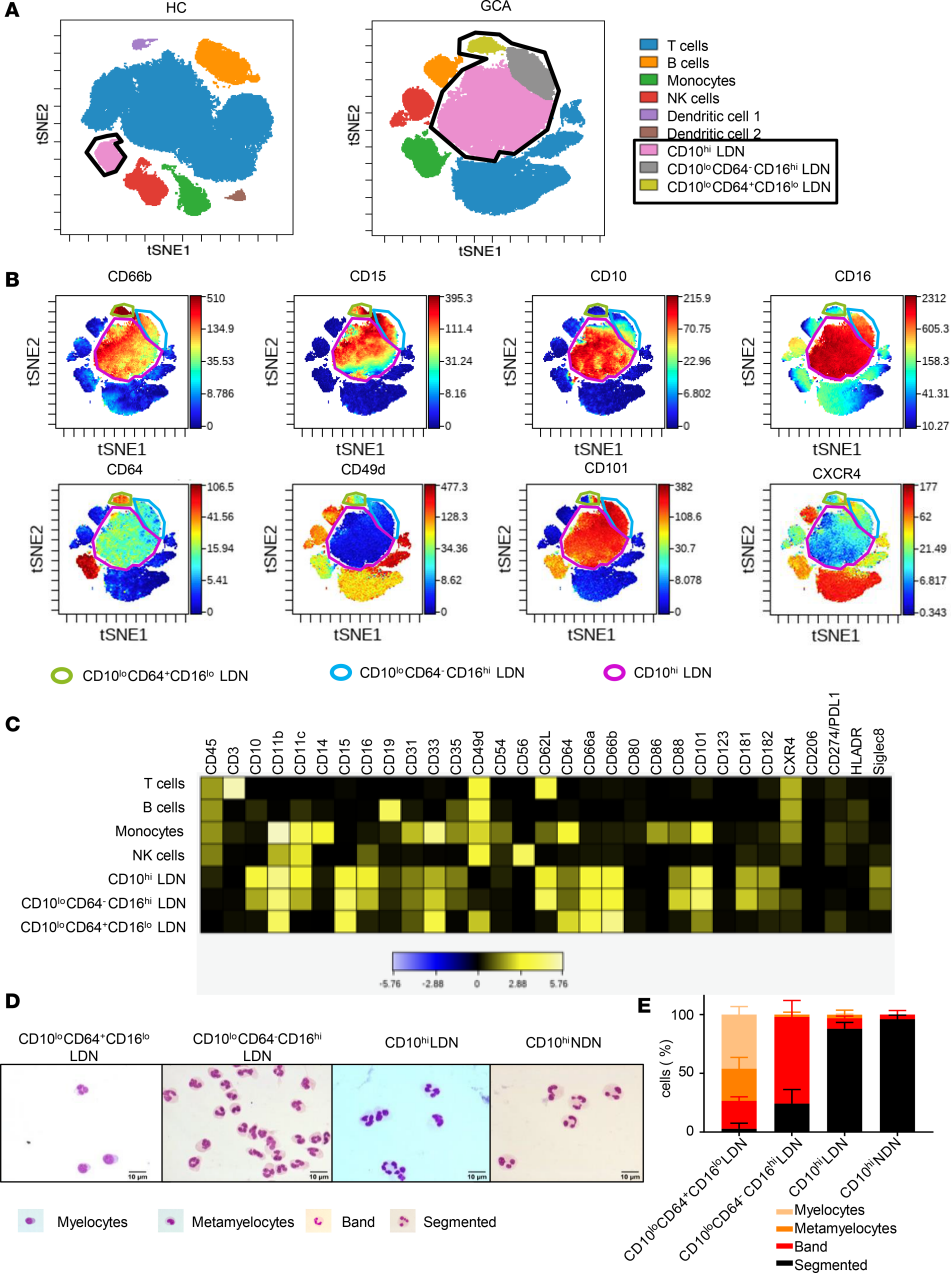
GCA patients are characterized by the presence of immature neutrophil populations in their blood. (**A**) Samples were clustered using viSNE, and cell populations were identified by expression of the main canonical markers. Representative viSNE clustering plots for 1 healthy control (HC) and 1 GCA PBMC sample are shown. Circled in black are total low density neutrophils (LDNs). (**B**) Highlighted expression of key neutrophil surface markers on viSNE plots of PBMC from 1 representative GCA. (**C**) The expression levels of selected markers in each of the identified cell populations are shown in the expression heatmap from 1 representative GCA patient. (**D**) Wright-Giemsa staining of FACS purified 4 neutrophil populations. One representative from at least 3 independent experiments of 3 GCA patients is shown. (**E**) Quantification of neutrophil of different maturation stages in each LDN population showed that, while both CD10^hi^ LDNs and NDNs were predominantly made of mature segmented neutrophils, myelocytes and metamylocytes contributed 80% of CD10^lo^CD64^+^CD16^lo^ LDNs, and 80% immature band neutrophils were found in CD10^lo^CD64^–^CD16^hi^ LDNs. Data are presented as mean ± SD.

To identify specific and reliable cellular markers of GCA, we employed mass cytometry (CyToF) analysis to peripheral blood mononuclear cell (PBMC) recovered during Ficoll density centrifugation, from newly diagnosed or flaring patients with active GCA and healthy controls (HCs). At the time of recruitment, patients with GCA received either no steroid treatment or a short period of low-dose treatment to control their flare (Supplemental Table 1 for CyToF antibody panel and Supplemental Table 2 for patient demographics and clinical features; supplemental material available online with this article; https://doi.org/10.1172/jci.insight.139163DS1). Two-dimensional reduction of viSNE (a visualization tool for high-dimensional single-cell data) analysis revealed the emergence of 2 CD66b^+^CD15^+^ neutrophil populations in PBMCs of patients with GCA that were not detected in HCs (Figure 1A). The 2 GCA neutrophil populations were characterized by low or no expression of CD10 and further split by the expression of CD64 (Figure 1, A and B). The third neutrophil population, defined as CD66b^+^CD15^+^CD10^hi^CD64^–^, was expanded in GCA patients but also detected in HCs (Figure 1A).

Comparison of other key markers reported to be differentially expressed during neutrophil maturation — CD16 (28), CD49d (28, 29), CD101 (29), and CXCR4 (30) — indicated that the 2 CD10^lo/–^ populations were likely to represent distinct populations of immature neutrophils (31) (Figure 1B). CD66b^+^CD15^+^CD10^lo/–^CD64^–^ LDNs were CD16^hi^CD49d^–^CD101^hi^CXCR4^lo/–^, whereas CD66b^hi^CD15^+^CD10^lo/–^CD64^+/bright^ LDNs were CD16^lo^CD49d^+^CD101^lo^CXCR4^lo/–^. For simplicity, LDNs populations were termed as CD10^hi^, CD10^lo^CD64^–^CD16^hi^, and CD10^lo^CD64^+^CD16^lo^ LDNs. Their distinct phenotypes were further confirmed by comparing major myeloid and lymphoid cell surface markers with other immune cells in PBMC (Figure 1C).

Total circulating neutrophils were subsequently separated into normal-density neutrophils (NDNs) and LDNs by Ficoll density gradient centrifugation and flow cytometry (FACS) sorting using the antibodies to CD66b, CD15, CD16, CD10, and CD64 (Supplemental Figure 1A and Supplemental Table 4). NDNs expressed high levels of CD66b, CD15, CD16, and CD10 and had no expression of CD64, phenotypically resembling CD66b^+^CD15^+^CD10^hi^CD64^–^ LDNs, and they were consequently termed CD10^hi^ NDNs. Morphology analysis of the 4 FACS purified neutrophil populations indicated that both CD10^hi^ NDNs and CD10^hi^ LDNs consisted largely of mature segmented neutrophils (Figure 1, D and E). CD10^lo^CD64^–^CD16^hi^ LDNs morphologically resembled immature band neutrophils, whereas CD10^lo^CD64^+^CD16^lo^ LDNs consisted up to 80% of myelocytes and metamyelocytes (Figure 1, D and E). The mapped populations of circulating neutrophils in the blood of vasculitis patients phenotypically resembled human BM neutrophils at different differentiation stages (Supplemental Figure 1B).

### CD10^lo^ LDNs with extended life span are clinically relevant to GCA.

**Figure 2:**
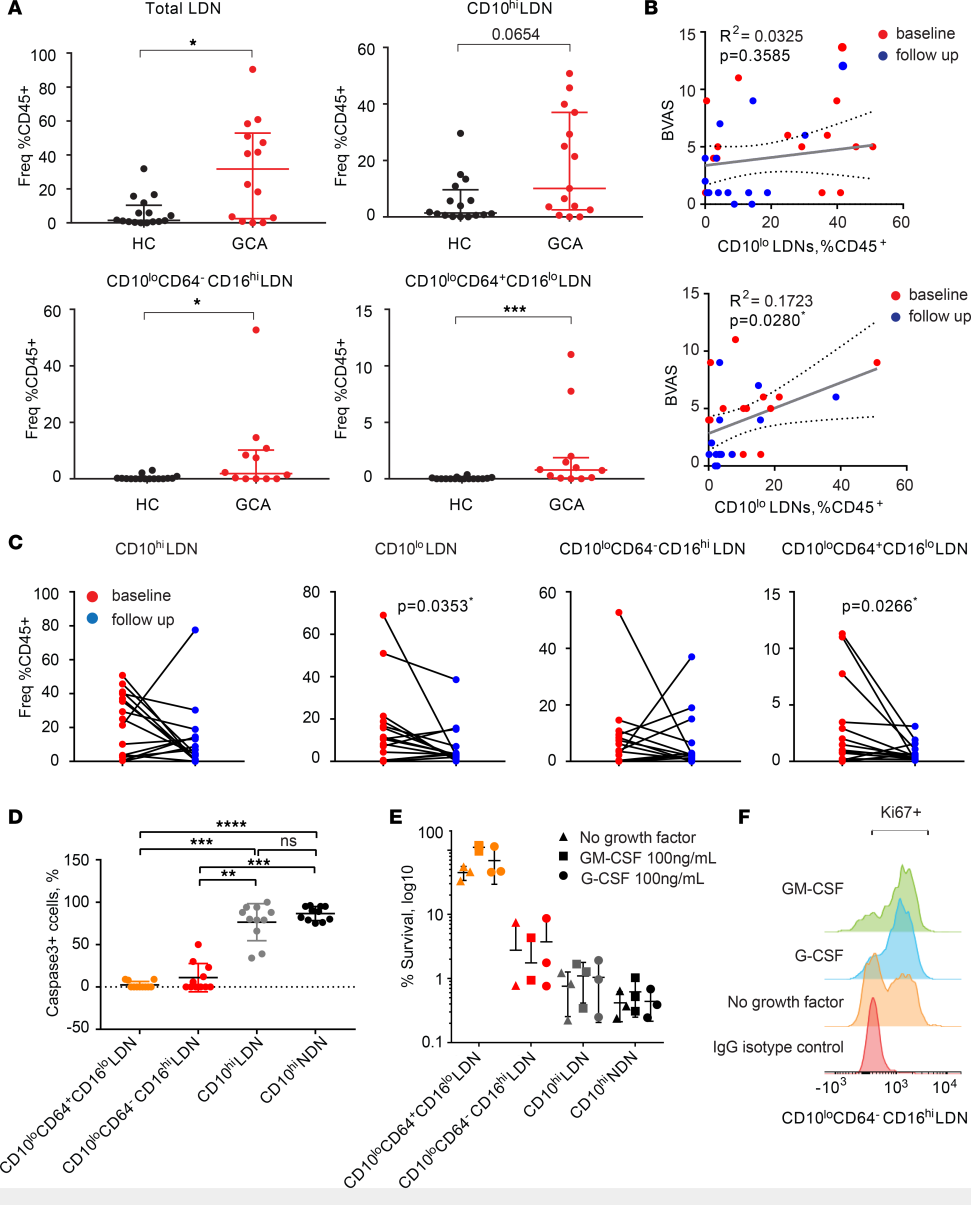
CD10^lo^ LDNs with extended life span are clinically relevant to GCA. (**A**) Neutrophil population frequency comparison between GCA and HC (GCA, *n* = 14; HC, *n* = 16). Unpaired nonparametric Mann-Whitney *U* test was used for statistical analysis. Data are presented with median ± IQR. (**B**) Correlation of CD10^hi^ and CD10^lo^ LDNs at baseline and follow-up after a single high-dose prednisolone treatment. Spearman’s correlation coefficient was calculated (baseline, *n* = 13; follow-up, *n* = 13). (**C**) Correlation of neutrophil subset frequency at baseline and follow-up. CD10^lo^CD64^+^CD16^lo^ and total CD10^lo^ LDNs showed significant negative correlation. A nonparametric paired Wilcoxon test was used for statistical analysis to compare the difference of neutrophil populations between baseline and follow-up measurement within the same patients. (**D**) The apoptotic rate of immature CD10^lo^CD64^–^CD16^hi^ and CD10^lo^CD64^+^CD16^lo^ LDNs was significantly reduced compared with mature CD10^hi^ NDNs and LDNs after 24 hours in vitro culture as evidenced by caspase-3 staining (no growth factor in presence). Quantification of caspase-3–activated cells was performed by counting FITC^+^ out of the total DAPI^+^ cells. Three independent experiments were performed on FACS purified neutrophil populations from 3 GCA patients. The quantification was carried out by counting cells from 4–5 fields of a total of 100–200 cells of each population of each donor. Data are presented as mean ± SD. Nonparametric 1-way ANOVA Kruskal-Wallis test was used for statistical analysis. (**E**) CD10^lo^CD64^+^CD16^lo^ LDNs could survive up to 5 days in vitro, even without the presence of neutrophil growth factors such as G-CSF and GM-CSF. (**F**) CD10^lo^CD64^+^CD16^lo^ LDNs retained the proliferation capacity up to 3 days in culture measured by Ki67 expression, which is associated with cellular proliferation. Histograms from 1 representative GCA donor are shown. Three independent experiments were performed on FACS purified neutrophil populations from 3 GCA patients in **E** and **F**. *P ≤ 0.05, **P ≤ 0.01, ***P ≤ 0.001, ****P ≤ 0.0001.

While patients with GCA showed a global increase in both total LDN and neutrophils (Figure 2A and Supplemental Figure 2A), the CD10^lo^CD64^–^CD16^hi^ and CD10^lo^CD64^+^CD16^lo^ populations were only detected in the GCA samples — not in HCs (Figure 2A). Similar results were obtained for the patients with GPA with active disease (Supplemental Figure 2B). The frequency of the CD10^lo^ but not CD10^hi^ population in the PBMCs correlated with clinical disease activity, as measured using version 3.0 of the Birmingham Vasculitis Activity Score (BVAS) (Figure 2B). Moreover, we observed a significant reduction in the frequency of total CD10 ^lo/–^ and CD10^lo^CD64^+^CD16^lo^ populations in GCA after 12–16 weeks of treatment with prednisolone (Figure 2C).

In contrast to mature CD10^hi^ NDNs and CD10^hi^ LDNs, CD10^lo^CD64^–^CD16^hi^ and CD10^lo^CD64^+^CD16^lo^ LDNs showed reduced rates of apoptosis after 24 hours in in vitro culture (Figure 2D). Consequently, 45% of CD10^lo^CD64^+^CD16^lo^ LDNs and 8% CD10^lo^CD64^–^CD16^hi^ LDNs were alive after 5 days in in vitro culture without any growth factor (Figure 2E). Moreover, CD10^lo^CD64^+^CD16^lo^ LDNs were still proliferating at day 5 in in vitro culture (Figure 2F). Their survival and proliferation rates were further potentiated in the presence of growth factors, especially GM-CSF (Figure 2, E and F). In addition, CD10^lo^CD64^+^CD16^lo^ LDNs had the potential to further differentiate into a more mature state, as they exhibited increased CD10 and CD16 expression after 5 days of in vitro culture with G-CSF (Supplemental Figure 2C). Together, these data suggest that the immature neutrophil populations remained in the circulation of GCA patients for an extended period of time and were associated with clinical disease activity.

### Immature neutrophils extravasate into temporal artery walls of GCA patient biopsies.

**Figure 3:**
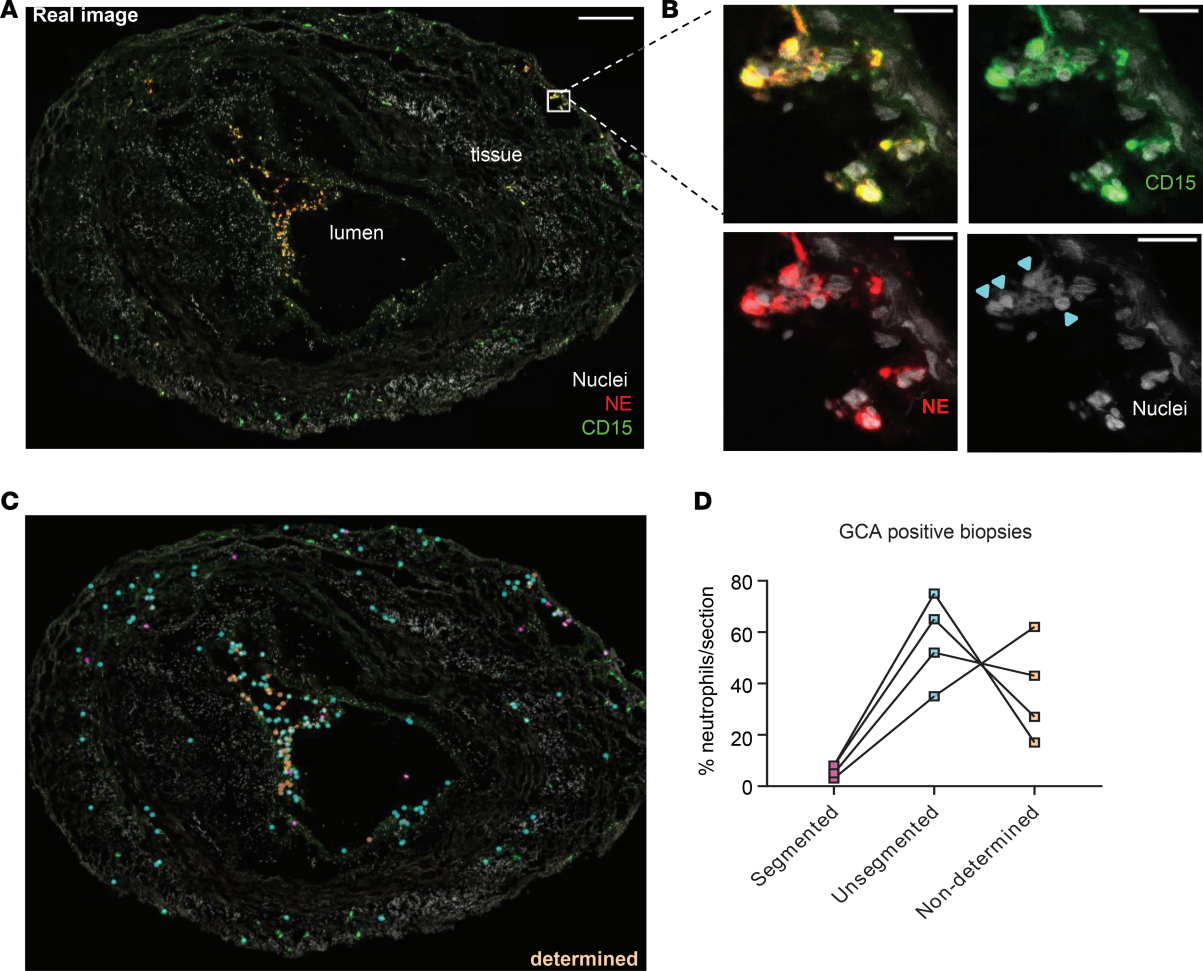
Immature neutrophils extravasate into temporal artery walls of GCA patient biopsies. (**A**) Confocal image of a temporal artery section of a GCA biopsy stained for neutrophil Elastase (NE, red), CD15 (green), and Hoechst (gray) for DNA revealed presence of neutrophils in both the lumen and tissue. Scale bar: 200 μm. (**B**) Zoomed-in regions in a gallery overview exemplify the morphology of both segmented and unsegmented nuclei within the specimen and emphasize the individual staining (from left to right and top to bottom: all combined, Hoechst + CD15, Hoechst + NE, Hoechst-only nuclei). Arrowheads indicate unsegmented neutrophil nuclei. Scale bar: 20 μm. (**C**) The point map of the whole section shows categorization of neutrophils based on the nuclear shape. (**D**) The respective quantification of neutrophils on 1 section from 4 different biopsies.

In the vasculature, neutrophils continuously patrol for activated platelets to initiate inflammatory responses (32). Indeed, we observed 15%–40% of LDNs from patients with GCA, especially CD10^lo^CD64^+^CD16^lo^ LDNs, costained with platelet glycoprotein Ib α chain (or CD42b), indicative of neutrophil-platelet aggregate formation (Supplemental Figure 3A). In contrast, CD10^hi^ NDNs of patients with GCA or HCs associated with very little CD42b (Supplemental Figure 3A). Neutrophil interactions with platelets facilitate their transmigration across the endothelium into the surrounding tissue (32). We therefore investigated if immature neutrophils could reach tissues surrounding temporal arteries of patients with GCA by an immunofluorescence (IF) analysis with antibodies against neutrophil elastase (NE) and CD15, expressed on all neutrophil populations. Consistent with previous reports (33), we observed few mature segmented neutrophils in temporal artery biopsies of GCA patients. However, NE and CD15 signals colocalized in the cells with nuclear morphology of immature neutrophils (Figure 3, A–C). Quantification of mature and immature neutrophils in the diseased samples (marked as segmented and unsegmented, respectively) confirmed the predominance of immature neutrophils in vascular tissues in GCA (Figure 3D and Supplemental Figure 3B). Immature neutrophils were found mainly in the lumen close to the internal elastica lamina and adventitia close to external lamina elastica but were largely absent in the media (Supplemental Figure 3C).

### CD10^lo^ LDNs are potent ROS producers but deficient in some innate immune functions.

**Figure 4:**
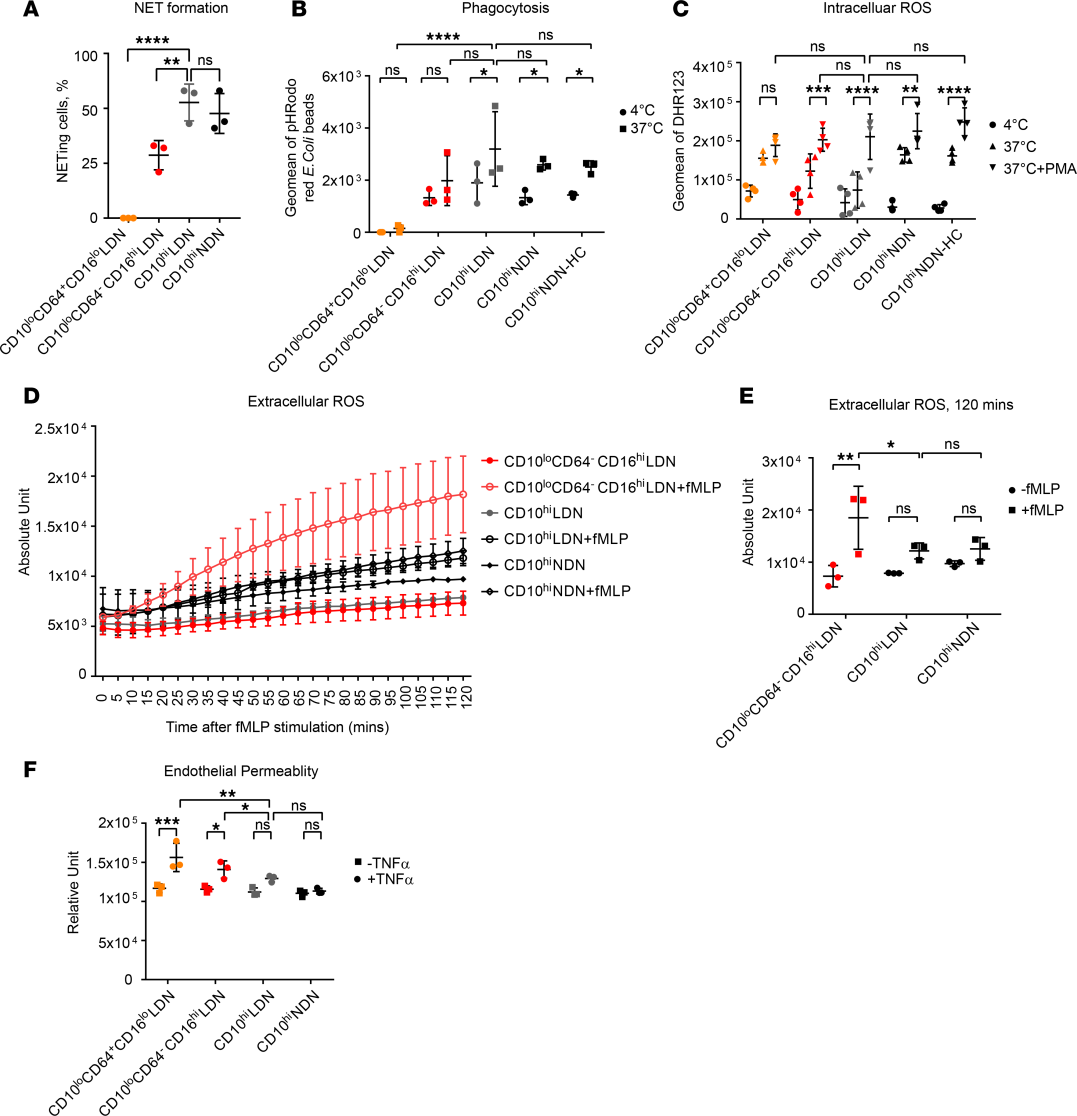
CD10^lo^ LDNs are potent ROS producers but deficient in some innate immune functions. (**A**) NET quantification by counting NET-forming cells stained positively with Cit-H3 and NE out of total DAPI^+^ cells. Three independent experiments were performed on FACS purified neutrophil populations from 3 GCA patients. (**B**) Phagocytosis was FACS quantified by the intake of pHrodo red *E. coli* bioparticles across the neutrophil populations. Mature but not immature neutrophils were capable of efficient phagocytosis. Three independent experiments were performed on FACS purified neutrophil populations from 3 GCA patients. (**C**) Intracellular ROS production was FACS quantified by green fluorescence converted from dihydrorhodamine 123 (DHR123) in the presence of ROS. Both mature and immature LDNs could generate ROS intracellularly, comparable with mature NDNs under PMA stimulation. Four independent experiments were performed on FACS purified neutrophil populations from 4 GCA and 4 HC donors. (**D**) Extracellular ROS was measured using OxyBURST Green H_2_HFF BSA. In the presence of 1 μM fMLP, immature CD10^lo^CD64^–^CD16^hi^ LDNs showed an increased and sustained release of ROS in comparison with CD10^hi^ LDNs and CD10^hi^ NDNs up to 120 minutes. (**E**) Extracellular ROS production at 120 minute with or without fMLP treatment. (**F**) Vascular damage was quantified by a permeability assay of endothelial barrier in a neutrophil-endothelial coculture system. Both CD10^lo^CD64^–^CD16^hi^ and CD10^lo^CD64^+^CD16^lo^ immature LDNs showed higher permeability compared with CD10^hi^ NDNs, indicating their potential roles associated with vascular damage in vasculitis. Three independent experiments were performed on FACS purified neutrophil populations from 3 GCA patients in **D**, **E**, and **F**. Two-way ANOVA was used for statistical analysis in **A**, **B**, **C**, **E**, and **F**. Data are presented as mean ± SD. *P ≤ 0.05, **P ≤ 0.01, ***P ≤ 0.001, ****P ≤ 0.0001.

Both NET release (34) and ROS production (35) have been intimately linked to vascular-damaging effects and injury. Thus, we examined innate immune functions of the 4 neutrophil populations. Only mature CD10^hi^ LDNs and NDNs efficiently formed NETs in response to ionomycin (Figure 4A and Supplemental Figure 4A) and also were capable of phagocytosing fluorescently labeled bioparticles conjugated with *E. coli* (Figure 4B), while immature LDN populations were partially or completely deficient in these effector functions. However, CD10^lo^CD64^–^CD16^hi^ LDNs produced comparable levels of free intracellular ROS to CD10^hi^ LDNs and NDNs in response to stimulation with phorbol myristate acetate (PMA) (Figure 4C). The levels of mitochondrial ROS, critical for spontaneous release of NETs made of oxidized mitochondrial DNA (36), were also comparable between these populations, in response to PMA (Supplemental Figure 4B). A close inspection of the data revealed that CD10^lo^CD64^+^CD16^lo^ LDNs displayed an elevated basal level of mitochondrial ROS production, which did not increase further with PMA stimulation.

We then studied the extracellular release of ROS, which is pertinent to vascular damage due to their direct effect on the endothelial cell barrier (35). We used OxyBURST Green H_2_HFF BSA, a sensitive fluorogenic protein conjugate, to detect extracellular release of oxidative products by neutrophil populations. Strikingly, immature CD10^lo^CD64^–^CD16^hi^ LDNs from patients with GCA generated a significant amount of extracellular ROS in response to stimulation with N-formyl-L-methionyl-L-leucyl-phenylalanine (fMLP), while mature CD10^hi^ LDNs and NDNs displayed limited production (Figure 4, D and E). Due to the low number of CD10^lo^CD64^+^CD16^lo^ LDNs, we could not test their capacity for extracellular ROS production in this assay. The potent release of extracellular ROS by immature CD10^lo^CD64^–^CD16^hi^ LDNs was mirrored by an increase in permeability of the endothelial barrier in the neutrophil-endothelial coculture system (Figure 4F). CD10^lo^CD64^+^CD16^lo^ LDNs were also capable of inducing endothelial barrier permeability (Figure 4F). We concluded that CD10^lo^ LDNs maintain intra- and extracellular ROS production, despite being deficient in some other innate immune functions.

### ROS biosynthesis pathways are active early in neutrophil development.

**Figure 5:**
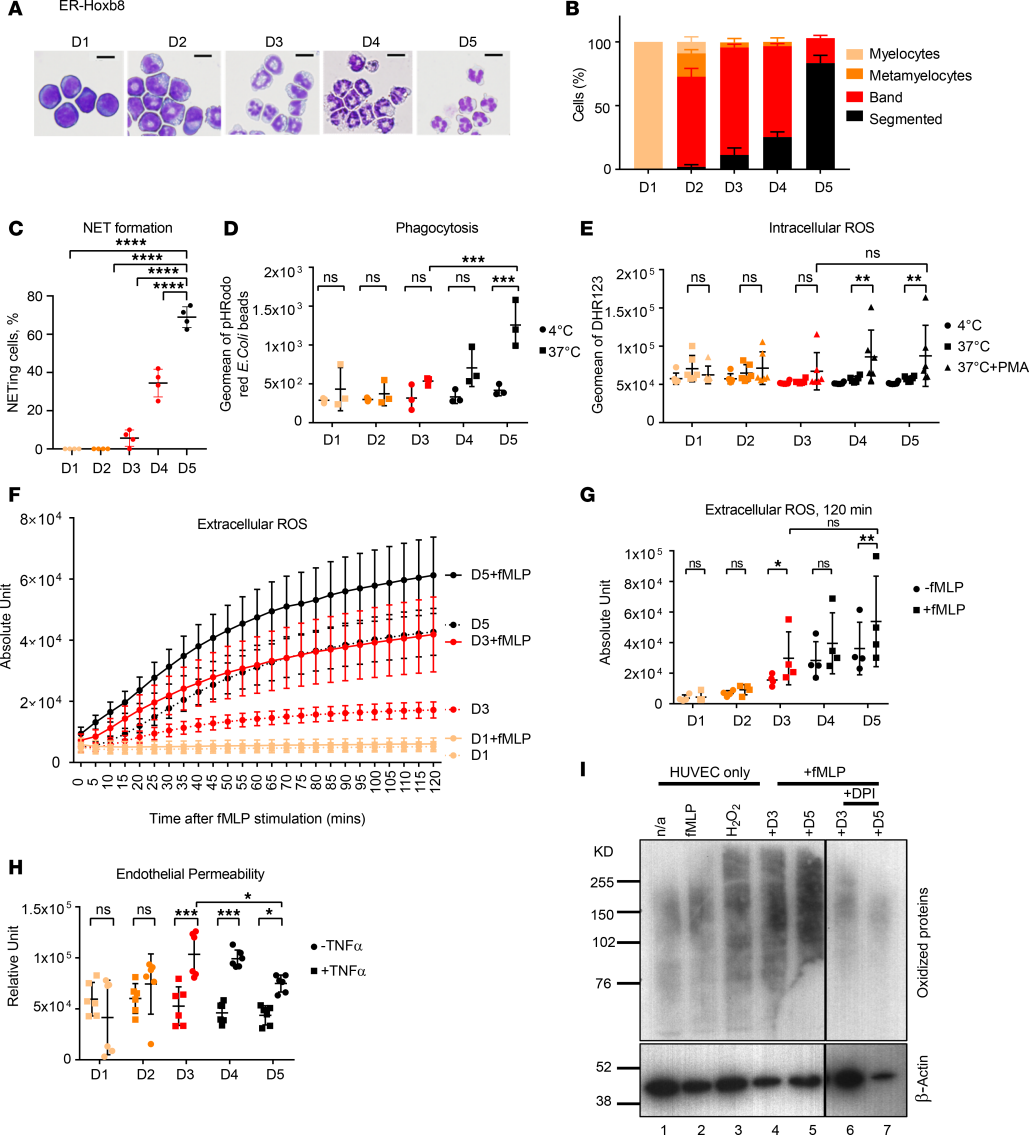
ER-Hoxb8–differentiated neutrophils faithfully recapitulate the phenotypes and functions of human neutrophil counterparts. (**A**) Morphology of ER-Hoxb8–differentiated neutrophils from D1 to D5 in the presence of G-CSF. Scare bars: 10 μm. One representative from 3–5 independent experiments is shown. (**B**) Quantification of neutrophil of different maturation stages in each ER-Hoxb8–differentiated neutrophil defined by days after G-CSF treatment. Data from at least 3 independent experiments are shown. (**C**) NET quantification by counting NET-forming cells stained positively with Cit-H3 and NE out of total DAPI^+^ cells. (**D**) Only mature D5 Hoxb8 neutrophils were able to perform phagocytosis. (**E**) Immature D3 Hoxb8 neutrophils were competent intracellular ROS producer comparable with mature D5 counterpart. (**F**) Immature D3 Hoxb8 neutrophils were competent to generate extracellular ROS over a period of 120 minutes. Five-minute intervals were used to plot each time point. (**G**) Extracellular ROS production at 120 minutes was compared across ER-Hoxb8 neutrophils of different days of maturation. (**H**) D3 Hoxb8 neutrophils showed higher endothelial permeability compared with neutrophils of other maturation stages in neutrophil-endothelial coculture permeability system. Data from at least 3 independent experiments are shown from **C** to **H**. (**I**) Detection of oxidized protein in HUVECs with Hoxb8 neutrophil coculture. Oxidized proteins potentially caused by extracellular ROS generated by D3 and D5 Hoxb8 neutrophils in the presence of 1 μM fMLP were detected via carbonylated groups in HUVEC. HUVEC without any treatment (–fMLP) and with fMLP alone (+fMLP) were used as negative controls. HUVECs treated with 10 μM H_2_O_2_ were used as the positive control. One representative from 3 independent experiments is shown. Two-way ANOVA was performed for statistical analysis in **C**, **D**, **E**, **G**, and **H**. Data are presented as mean ± SD. *P ≤ 0.05, **P ≤ 0.01, ***P ≤ 0.001, ****P ≤ 0.0001.

To better understand the molecular mechanisms driving ROS production by immature LDNs and to control for differences in the environment, we used an ex vivo system of murine homebox oncoprotein Hoxb8 driven by estrogen receptor (ER-Hoxb8) neutrophils that phenotypically and morphologically recapitulate neutrophil maturation stages during 5 days of culture in the presence of G-CSF. Morphology assessment and analysis of key lineage markers demonstrated that CD10^lo^CD64^+^CD16^lo^ LDNs are most similar to Hoxb8 neutrophils at day 1 (D1)/ D2 of their ex vivo differentiation, CD10^lo^CD64^–^CD16^hi^ LDNs to D3/D4, and fully mature CD10^hi^ LDNs and NDNs to D5 (Figure 5, A and B; and Supplemental Figure 5A). Only D5 Hoxb8 neutrophils could form NETs potently (Figure 5C and Supplemental Figure 5B) and phagocytose (Figure 5D), but both D5 and D3 neutrophils were competent intracellular (Figure 5E) and extracellular (Figure 5, F and G) ROS producers, consistent with the recently reported analysis of human BM neutrophils (37). In support of our findings with CD10^lo^ LDNs, the release of extracellular ROS by D3 and D5 Hoxb8 neutrophils led to permeability of the endothelial barrier in the coculture system (Figure 5H). To confirm the direct effect of neutrophil-produced ROS on protein oxidation, we measured protein carbonylation in the endothelial cells from the coculture system. D3 and D5 Hoxb8 neutrophils triggered a similar level of this irreversible oxidative protein modification as hydrogen peroxide treatment (Figure 5I), indicating significant oxidative stress caused by immature neutrophils. Blocking of extracellular ROS production by neutrophils with the NADPH oxidase inhibitor diphenyleneiodonium (DPI) (Supplemental Figure 5C) led to amelioration of neutrophil-induced endothelial leakage and the occurrence of oxidized proteins (Figure 5I and Supplemental Figure 5D).

**Figure 6:**
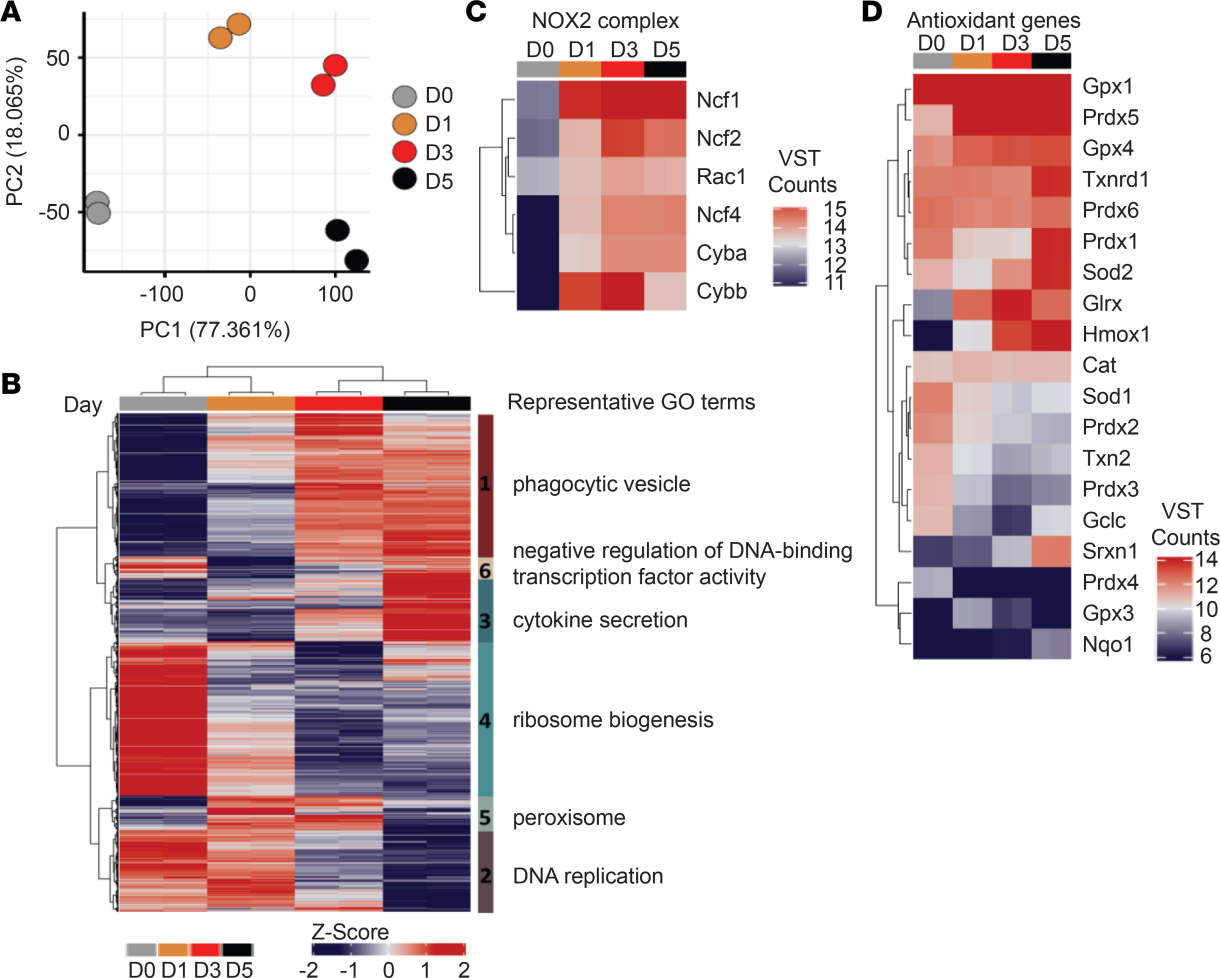
Immature neutrophils display functional ROS biosynthesis system, but the antioxidant systems develop with maturation. (**A**) The first 2 components from principle component analysis (PCA) of 11,412 differentially expressed (adjusted *P* < 0.01) are shown, which separate the cells by maturity. (**B**) Z-scores of differentially expressed genes (as in **A**). Hierarchical clustering of Euclidean distances reveals 6 distinct clusters, which differ in their pattern of expression across differentiation. Representative gene ontology (GO) terms are shown for each cluster. (**C** and **D**) Heatmaps of variance stabilized counts for differentially expressed genes comprising the NADPH oxidase 2 (NOX2) complex (**C**) and selected antioxidant genes (**D**).

To investigate the molecular mechanisms of the ROS production in immature neutrophils, we compared transcriptomes of D0, D1, D3, and D5 Hoxb8 neutrophils by RNA sequencing (RNA-seq). Principle component analysis (PCA) of differentially expressed genes (*P* < 0.05) clearly separated all stages of development (Figure 6A). Gene ontology (GO) analysis of 11,412 differentially expressed genes across the Hoxb8 neutrophil maturation revealed that they encompassed a number of core neutrophil processes, including phagocytosis and cytokine production, which peaked at D3 and D5, respectively (Figure 6B). Of relevance, biosynthesis of oxidative organelle peroxisome, important for conversion of ROS, peaked already between D1 and D3 (Figure 6B). In phagocytes, NADPH oxidase complex 2 (NOX2) is the enzyme responsible for the generation of superoxide, which can be converted to various ROS, including hydrogen peroxide. NOX2 complex is composed of cytochrome b α (CYBA) and β (CYBB) chains. CYBB (gp91-phox) also peaked at D1 and D3 in Hoxb8 neutrophils, consistent with previously reported expression in immature neutrophils in both human and mouse BM (29, 37) (Figure 6C). However, a number of ROS biosynthesis-related genes and antioxidant system enzymes responsible for destroying free superoxide radicals in the body (e.g., superoxide dismutase [Sod], peroxiredoxin [Prdx], sulfiredoxin [Srxn], thioredoxin reductase [Trxn] catalase [Cat], glutathione peroxidase [Gpx]) peaked at D5, including *Prdx1*, *Hmox1*, *Sod2*, *Txnrd1*, and *Srxn1* (Figure 6D). Analysis of recently published human BM neutrophil gene expression data (37) also showed higher levels of expression for a number of antioxidant enzymes, such as *GPX1,3,4*; *SOD2*; *HMOX2*; *TXNRD1*; and *SRXN1* in segmented mature neutrophils (Supplemental Figure 6A). A close look into the transcriptome of enriched CD10^+^ and CD10^–^ LDNs from SLE patients published by Mistry et al., revealed a highly similar pattern of increased expression of *SOD2*, *HMOX2*, *SRXN1*, and *TXNRD1* in mature CD10^+^ LDNs (38) (Supplemental Figure 6, B and C).

Together, it appears that, while ROS biosynthesis pathways are active early in neutrophil development, the antioxidant system appears to fully develop only in fully mature neutrophils, providing some possible explanation to the high level of extracellular ROS observed in CD10^lo^ LDNs.

## Discussion

Though not usually life threatening, GCA has serious long-term morbidity (especially visual loss) with poor patient outcomes if not diagnosed and treated early. The histological hallmark of GCA is formation of multinucleated giant cells at the intima-media junction (present in around 70% of biopsies). Other typical immunopathological features of GCA include infiltration of T lymphocytes, macrophages, and monocytes but not B cells in the lesion. In the present study, we endeavored to comprehensively map neutrophil populations at the disease onset, dissect their functions, and reveal their potential roles in GCA pathogenesis and application in clinical signature development and therapeutic intervention.

Applying single cell CyToF to PBMC isolated from blood samples of GCA, we have identified 4 neutrophil populations that are phenotypically described to display a continuum of maturation from precursor myelocytes to mature segmented neutrophils. We found that CD10 together with CD64 clearly delineated the maturation stages of the neutrophil population, with CD10^lo^CD64^–^CD16^hi^ LDNs morphologically resembling immature band neutrophils and CD10^lo^CD64^+^CD16^lo^ LDNs resembling myelocytes and metamyelocytes (Figure 1). As expected, immature CD10^lo^CD64^+^CD16^lo^ and CD10^lo^CD64^–^CD16^hi^ LDNs showed extended life span in vitro compared with mature CD10^hi^ LDNs and NDNs. In addition, CD10^lo^CD64^+^CD16^lo^ LDNs strikingly retained proliferation capabilities in vitro, which might result in their extended life span (Figure 2). In the presence of G-CSF, CD10^lo^CD64^+^CD16^lo^ LDNs acquired the expression of CD10 and CD16 after 5 days in in vitro culture, suggesting this subset of neutrophils were fully competent precursor neutrophils (Supplemental Figure 2).

In contrast to previous studies (20), we have found LDNs to be present in both vasculitis patients and HCs, although GCA patients had a higher LDN counts. It was the immature CD10^lo^ LDNs that were rarely present in health and correlated with GCA disease score (Figure 2). Of interest, the opposite correlation was observed in patients with SLE, where the frequencies of CD10^+^ LDNs, but not CD10^−^ LDNs, correlated with the lupus damage index (38). In particular, immature CD10^lo^CD64^+^CD16^lo^ LDNs increased consistently in systemic vasculitis of both GCA and GPA. The cohort of patients with GCA used in this study was stratified for receiving either no treatment or a short-term, low-dose prednisolone treatment at recruitment. Thus, the known effect of glucocorticoids on the release of immature neutrophils in circulation (39) was very limited in this cohort. An independent study of 537 newly diagnosed GCA from Oh et al. also suggested that glucocorticoids are unlikely to be a confounding factor in the observed elevated neutrophil/lymphocyte ratio in active GCA patients (40). Importantly, we detected a significant reduction of immature CD10^lo^ neutrophils after prolonged prednisolone treatment (Figure 2C), likely reflecting on the underlying disease state, rather than glucocorticoid use itself (41). The correlation between BVAS and CD10^lo^ LDN frequencies (Figure 2B) also favors this scenario.

The unsegmented and monocyte-like nucleus of immature neutrophils could be the reason for them to remain undetected in GCA patient biopsies, despite extensive research (42). We found that predominantly immature CD15^+^NE^+^ neutrophils were indeed present in the temporal artery biopsies; they infiltrated into both lumen and artery walls of temporal arteries taken from patients with newly diagnosed GCA (Figure 3). Importantly, CD15^+^ neutrophils were also found in renal biopsies of GPA and ANCA-associated glomerulonephritis patients, where they were identified as a major source of established autoantigens — e.g., myeloperoxidase (MPO) (43–45). Therefore, our finding of immature neutrophils in the GCA biopsies offers clues to identify and investigate immature neutrophils in the initial vascular damage of AAVs, such as GPA.

Taken together, our findings support the hypothesis that immature CD10^lo^CD64^+^CD16^lo^ and CD10^lo^CD64^–^CD16^hi^ LDNs are bona fide neutrophil subsets associated with systemic vascular inflammation.

CD10 has recently been proposed as the key marker to differentiate immature neutrophils from mature neutrophils of both LDN and NDN fractions (31). The study used healthy donors treated with G-CSF, which is known to play a pivotal role to mobilize neutrophils from the BM. The sudden increase of G-CSF in the circulation of the treated donors mimics emergency granulopoiesis that prematurely releases immature neutrophils into the circulation. Of relevance, cancer patients on chemotherapy and G-CSF treatment can develop large-vessel vasculitis as a possible serious adverse event (46).

Neutrophil CD64 expression has been used as a diagnostic marker of infection and sepsis in the ICU (47) and was found to be superior to C-reactive protein and hematological determinations for detecting systemic infection or sepsis (48). However, these studies did not distinguish between the differential marker expression due to mature neutrophil activation or due to appearance of another neutrophil population. Because we observed no CD64 expression on mature neutrophils, either from NDN or LDN fractions in GCA (Figure 1), we hypothesize that detection of CD64 expression on neutrophils in sepsis is likely to represent the presence of CD10^lo^CD64^+^ myelocytes/metamyelocytes in the neutrophil pool. Coincidentally, in a recently deposited open-access manuscript, Meghraoui-Kheddar et al. used a similar single-cell CyToF strategy to identify 2 CD64^+^ neutrophil subsets unequivocally associated with an onset of sepsis (49). CD123 and PD-L1 were reported to be the additional and distinctive cellular markers for sepsis-induced CD64^+^ immature neutrophils compared with noninfectious inflammatory syndrome. Indeed, we observed no CD123 and very weak PD-L1 expression on CD64^+^ immature neutrophils of GCA patients (Figure 1), supporting a possible new demarcation between CD10^lo^CD64^+^ released in response to infection and sterile inflammation.

Functionally, immature neutrophils displayed complete or partial loss of NET formation and phagocytosis, but they were capable producers of both intracellular and extracellular ROS (Figure 4). Their active extracellular ROS production reported enhanced degranulation (38), and interaction with platelets underlined immature neutrophils’ ability to cause significant vascular damage in vitro to endothelial cell monolayer of HUVECs, as evidenced by increased cell permeability (Figure 4). Taken together with the observation that immature neutrophils infiltrated into the artery cell wall, it is possible that CD10^lo^ LDNs may be directly involved in GCA pathogenesis.

The ER-Hoxb8 differentiated neutrophils faithfully recapitulated the phenotypes and functions of human neutrophil subsets at respective maturation and differentiation stages (Figure 5).

We exploited this ex vivo cell system (50, 51) to map the gene circuits in control of ROS homeostasis in neutrophils. By comparing our data with published transcriptome data sets from human BM neutrophils and purified SLE LDN populations, we have found that immature neutrophils D1/D3 Hoxb8, human BM metamyelocytes, band neutrophils, and CD10^–^ SLE LDNs all exhibited high CYBB expression, which suggests an enhanced capacity for superoxide production. Since superoxide is highly toxic to the cell, complex molecular networks of antioxidants are engaged to convert it to other ROS, such as H_2_O_2_, which are further reduced to H_2_O. When cells fail to scavenge excessive ROS, H_2_O_2_ and other ROS can diffuse directly to extracellular space and irreversibly damage neighboring cells. Important and common antioxidant genes include *SOD*, *CAT*, and different variants of *PRXD* and *GPX* genes. While many of these genes showed similar expression between immature and mature neutrophils from Hoxb8 neutrophils, human BM neutrophils and SLE LDN — some, such as *SRXN1*, *TXNRD1*, *SOD2*, and *HMOX1/2*, were highly expressed only in mature neutrophils from mouse to human (Supplemental Figure 5). Thus, the large amounts of ROS generated by immature neutrophils in response to inflammatory signals may not be efficiently cleared due to incomplete antioxidant systems. When excessive extracellular ROS is released by the immature neutrophils (Figure 4), it may inadvertently cause tissue damage observed in GCA patients. Interestingly, Srxn-Prdx axis in ROS homeostasis have been implicated in cancer progression (52). This will be an interesting target for in-depth investigation on its role to control ROS production in immature neutrophils.

Herein, we identified and characterized 2 subsets of immature CD10^lo^ LDNs present in GCA with increased numbers, which inflicted oxidative damage to the endothelium. Moreover, we linked the active therapy to the disappearance of the respective neutrophil subset from GCA patient’s circulation. Due to the small sample size in our study, substantial effort will be needed toward development of CD10^lo^CD64^+^CD16^lo^ and/or CD10^lo^CD64^–^CD16^hi^ neutrophils as robust diagnosis and prognosis biomarkers. Large-scale screening is needed for their presence in the peripheral blood, coupled with IHC of the arterial vessel walls of patient biopsies. Furthermore, no appropriate animal models of GCA are currently available to mimic the clinical process of disease initiation and progression, though functional models have been developed to directly study arterial and large-vessel damage and inflammation (8). This has hindered our understanding of exact pathophysiological roles of immature neutrophil in GCA. An alternative can be the ex vivo 3D tissue culture system using the diseased artery tissue as developed by Corbera-Bellalta et al. (53), which will be useful to dissect the precise cellular mechanisms of neutrophil subsets in shaping the disease.

To conclude, we propose that immature neutrophils may play an active role in GCA pathogenesis. Through their extended life span and capabilities to cause vascular damage, immature neutrophils are the likely culprits to initiate blood vessel lesion, leading to local inflammation and — gradually — systemic vasculitis. Compared with macrophages and T cells, neutrophils were found to be present mainly in the lumen and adventitia but largely absent in the media of affected vessels (Supplemental Figure 3), suggesting that neutrophils are not directly involved in giant cell or granuloma formation. Therefore, we speculate a 3-stage model of neutrophils in GCA pathogenesis. Stage 1: ongoing chronic inflammation in patients causes release of immature neutrophils into the circulation with an extended life span. Stage 2: immature neutrophils enter from both lumen and capillaries and adhere to the elastic lamina for prolonged periods of time to release ROS in an inflammatory microenvironment, which results in the accumulation of small breaks and lesions in elastic lamina. Stage 3: other immune cells, including macrophages, monocytes, T cells, and DCs, infiltrate into the vessels via the initial lesions and gradually lead to formation of giant cells and/or granulomas culminating in severe vessel inflammation.

Based on this model, a potential therapeutic intervention in GCA, as well as other vasculitides, may be to suppress neutrophil ROS generation, using for example inhibitors of NADPH oxidases (54). Likewise, introducing antioxidants in anti-GCA armamentarium, such as the recently described ultrasmall copper-based nanoparticles (55) or fullerene-based antioxidant (56), which partly restored function in a primate model of Parkinson’s disease, may help patients to achieve a sustained remission and potentially reduce the use of glucocorticoid for improved quality of life.

## Methods

Supplemental methods are available online with this article.

### Patient recruitment, demographics, and ethnical approval.

Newly presenting patients or flaring patients of GCA were diagnosed with evidence of a positive halo on ultrasound of at least 1 temporal or axillary artery or a temporal artery biopsy showing features of active inflammation. BVAS and the Vasculitis Damage Index (VDI) were also assessed in all patients to assist in diagnosis and treatment responses. Both BVAS and VDI are validated tools for clinical evaluation across a range of systemic vasculitides (57, 58). In addition, the following criteria were observed to exclude patient from being recruited into the study: (a) previous diagnosis of RA or any inflammatory arthritis, or any form of vasculitis (apart from GPA or GCA); (b) long-term (>1 month), high-dose (>20 mg per day) steroid use, within 3 months before study entry, which results in substantial improvement in patients’ condition at the time of recruitment into the study; and (c) the presence of active infection associated with an increased CRP and WBC count. For newly presenting patients or flaring patients with GPA, a BVAS score of at least 3 was used as recruitment criteria. Written informed consent was obtained for each participant of the study. Demographics of both patients and HCs, and clinical characteristics of the patients, are summarized in Supplemental Table 2, A and B, including sex and age, while ethnicity information was excluded from this study by the decision of the investigators. For follow-up studies, all GCA patients received > 40 mg per day of prednisolone for 2–4 weeks, followed up by 5–10 mg per day for the remaining time, and had an excellent treatment response during the time-course of the study. Due to the restriction imposed on our patient and healthy volunteer recruitment, there was significant age gap (10 years) between the GCA patients and HCs. In the HC group, the majority of the donors were female (77%), while 43% of GCA patients were female. Therefore, we performed 2-way multivariate ANOVA (MANOVA) and used Pillai’s trace statistic to assess significance of each parameter using SPSS (SPSS version 24.0). We confirmed that age and sex are not confounding factors for the difference of neutrophil populations observed between GCA and HCs (Supplemental Table 3). Furthermore, a corrected model was also performed to adjust age and sex influence on the neutrophil populations (Supplemental Table 3), which also showed that age and sex have no significant effect on the neutrophil subset frequencies.

### TABUL samples.

The inflamed arteries used in the study were collected via temporal artery biopsy and ultrasound (TABUL) in diagnosis of GCA. TABUL is a multicenter study to compare temporal artery biopsy and ultrasound in diagnosis of GCA (4). As part of the TABUL protocol, all newly diagnosed patients were assessed using BVAS and VDI, and we continued to use these assessments in the course of this current study, in the absence of any disease-specific clinical tools to evaluate disease status in GCA.

### LDN and NDN isolation.

PBMC was isolated using lympholyte CL5020 based on the manufacturer’s instruction (Cedarlane Laboratories). Briefly, 10 mL of whole blood from patients or controls was collected by venipuncture with 10 mL lavender BD Vacutainer K2EDTA tubes and mixed with 10 mL HBSS buffer before being layered onto 20 mL Lympholyte-H in a 50 mL Falcon tubes. The Falcon tube was centrifuged at 500*g* for 20 minutes at room temperature (RT) with the brake off. Buffy coat was collected, topped with 40 mL HBSS buffer and centrifuged at 500*g* for 10 minutes at RT with the brake on to remove platelets. A total of 10 mL Ack lysis buffer (RBC cell lysis buffer; Thermo Fisher Scientific) was added to the cell pellet, incubated for 5 minutes at RT, and subsequently centrifuged at 500*g* for 5 minutes at RT to remove RBCs. The cell pellet was resuspended in 5 mL RPMI 1640 complete media (MilliporeSigma) for downstream experiments. LDNs were isolated and purified from PBMC by FACS based on the gating strategy illustrated in Supplemental Figure 1A.

NDNs were enriched by directly adding 20 mL of Ack lysis buffer to the bottom RBC layer after the buffy coat and lympholyte media were removed from the 50 mL falcon tube. Further purification of NDNs was performed by FACS based on the gating strategy illustrated in Supplemental Figure 1A.

### Cell lines.

Hoxb8 cells are murine myeloid progenitor cells isolated from transgenic mouse strains and immortalized with ER-Hoxb8 (51). Endogenous expression of Hoxb8 proteins enables the arrest of myeloid differentiation, resulting in an infinite progenitor expansion, which makes ER-Hoxb8–derived neutrophils an ideal model to study myeloid cell differentiation and investigate cellular functions of macrophages and neutrophils under normal or inflammatory conditions (50). The cells were a gift from the Walzog laboratory (University of Munich, Munich, Germany). ER-Hoxb8 progenitor cells were routinely cultured in RMPI-1640 medium supplemented with 10% FCS, 30 mM β-mercaptoethanol (Invitrogen), 1% supernatant from stem cell factor–producing (SCF-producing) CHO cells (provided by Hans Häcker, University of Utah, Salt Lake City, Utah, USA), and 1 μM β-estradiol (MilliporeSigma). Differentiation was induced by estrogen removal and culture in medium containing 1% SCF supernatant. The progenitor cells were differentiated into neutrophils by culturing with complete RPMI 1640 medium supplemented with 30 μM β-mercaptoethanol, 4% SCF containing supernatant, and 20 ng/mL G-CSF in 5% CO_2_ tissue culture incubator at 37°C.

HUVECs were obtained from the Monaco group (Kennedy Institute of Rheumatology, University of Oxford). Cells were cultured with EGM-2 growth medium (Lonza) in 5% CO_2_ tissue culture incubator at 37°C.

### CyToF.

Samples were first stained with rhodium DNA intercalator (Fluidigm) as an indicator of cell viability before Fc receptor blocking. Staining with metal-conjugated antibodies recognizing cell surface antigens (antibodies used for CyToF analysis were summarized in Supplemental Table 1) was performed for 30 minutes at RT before fixation of samples in 1.6% paraformaldehyde (Thermo Fisher Scientific) for 10 minutes. Finally, samples were stained overnight with iridium DNA intercalator (Fluidigm) in Maxpar fix and perm buffer (Fluidigm). Prior to acquisition, samples were washed twice with cell staining buffer and twice with Maxpar water (Fluidigm). Samples were resuspended in Maxpar water containing 10% EQ calibration beads (Fluidigm), filtered through a 40 μM cell strainer, and counted, and the concentration was readjusted to 0.5 × 10^6^ cells/mL for acquisition on Helios mass cytometer (Fluidigm). Resulting .fcs files were normalized using the normalization tools within the Helios software and uploaded to Cytobank (https://www.cytobank.org/) for all gating and further analysis. The dimensionality reduction algorithm viSNE, part of the Cytobank package, was used for clustering and visualization of the data. All directly conjugated antibodies used in the study were purchased from Fluidigm. Purified unlabeled antibodies were purchased from various suppliers: BioLegend, BD biosciences, and eBioscience (refer to Supplemental Table 1) and labeled using the Maxpar X8 Metal Labelling Kit (Fluidigm) according to the manufacturer’s instructions.

### Flow cytometry.

Flow cytometry acquisition and cell isolation were performed with a BD LSR II or Fortessa X-20 and FACSAriaII, respectively (BD Biosciences). Data analysis was performed using FlowJo v10 (Tree Star Inc.). Antibodies used to stain the cells are summarized in Supplemental Table 3.

### Cytospin.

A total of 5000–50,000 FACS purified human neutrophils or cultured ER-Hoxb8–derived neutrophils were collected and immobilized to glass microscope slides by centrifugal forces using a Cytospin 3 Cytocentrifuge (Shandon, Thermo Fisher Scientific) at 400*g* for 5 minutes. The slides were then air dried and fixed with methanol for 10 minutes before being stained with ready-to-use modified Wright-Giemsa stain from MilliporeSigma (catalog WG16) for 5 minutes at RT. Images were obtained from stained slides under bright field using an Olympus BX51 Ostometric fluorescence microscope (Olympus).

### Apoptosis, survival, and proliferation.

Apoptosis was measured by imaging with NucView 488 Caspase-3 assay kit for live cells (Biotium). Sorted human neutrophil subsets were cultured in complete RPMI 1640 medium and incubated in in 5% CO_2_ tissue culture incubator at 37°C. After 24 hours, the culturing medium was replaced with fresh medium containing 5 μM NucView 488 substrate stock solution, and cells were incubated with substrate at RT for 30 minutes. Cells were then pelleted down, washed with PBS, placed on a glass slide, and imaged by an Olympus BX51 Ostometric fluorescence microscope with excitation at 485 nm.

Survival of sorted neutrophil subsets in vitro under different culture conditions was measured by CellTiter-Glo luminescent cell viability assay from Promega based on the manufacturer’s instruction.

Proliferation of sorted neutrophil subsets was measured by FACS with anti–human Ki67-APC (clone 20Raj1, catalog 17-5699-42, eBioscience) staining.

### IF of GCA biopsies.

For IF staining, snap-frozen temporal artery biopsies from TABUL were cut into 5 μM–thick sections and kept at –20°C until staining. Before staining, sections were air-dried vertically for 30 minutes, rehydrated in PBS for 2 minutes, and fixed in 1:1 ratio of chilled methanol and acetone for 30 seconds followed by 3 consecutive washes of 5 minutes in PBS. PAP Pen was used to define the border around the sections and provide a water-repellent barrier, keeping staining reagents localized on the sections. In brief, the staining was carried out as follows: tissue specimens were blocked for 1 hour in PBS containing 5% normal goat serum, 10% BSA, 0.3M glycine, and 0.05% Triton X-100. Sections were incubated with the primary NE antibody (NP57, Insight Biotechnology) diluted 1:75 in Carbo Free blocking solution with 0.05% Triton X-100 for 1 hour at RT, followed by 1-hour secondary antibody (goat anti-mouse, IgG-AF488, Invitrogen) staining in 1:150 dilution. Anti-CD15 antibody (AF647, R&D systems) in 1:60 was incubated on the sections overnight at 4°C. Each staining step was followed by 3 consecutive washing steps of 5 minutes in PBS with 0.05% Triton X-100. Hoechst (Thermo Fisher Scientific) was applied on the sections in 1:5000 in PBS for 5 minutes and removed with 3 PBS washes of 5 minutes. Finally, sections were rinsed in dH_2_O, excess liquid was removed, and Vectashield Vibrance Antifade mounting medium (Vector Laboratories) was used to mount the coverslip onto the slides. Slides were allowed to cure overnight before imaging at 40× magnification on an Axio Examiner upright microscope equipped with the ZEISS LSM 880 confocal system.

### NETosis.

To induce NETosis, sorted human neutrophil subsets or Hoxb8 neutrophils were seeded into an 8-well lab-TekII chamber slide (VWR international) coated with 2% poly-lysine (MilliporeSigma) at a volume of 100 μL at the density of 1 × 10^6^/mL. Neutrophils were stimulated with 100 ng ionomycin (MilliporeSigma) for 1 hour at 37°C in a 5% CO_2_ tissue culture incubator and were subsequently fixed with 4% paraformaldehyde (MilliporeSigma) in DPBS for 30 minutes at RT. Afterward, cells were treated with 1:2000 DAPI (Invitrogen) in DPBS for 5 minutes, washed 3 times with DPBS, and incubated with blocking buffer (2%BSA TBST) for 20 minutes. Following blocking, primary antibodies — rabbit anti–citrullinated histone 3 (ab5103, Abcam) and mouse anti–human/mouse elastase (ab21595, Abcam) at 1:300 dilutio — were added and incubated for 2 hours at RT or overnight at 4°C. Cells were washed with DPBS before adding secondary antibodies — mouse anti–rabbit DyLight 594–conjugated secondary antibody (Thermo Fisher Scientific) and rabbit anti–mouse IgG secondary antibody conjugated with Alexa Fluor-488 (Thermo Fisher Scientific) — at 1:300 dilution for a 1-hour incubation at RT in the dark and washed again with DPBS. Images were obtained by an Olympus BX51 Ostometric fluorescence microscope with excitation at 480 nm and emission 525 nm for green fluorescence, and excitation at 594 nm and emission at 610 nm for red fluorescence.

### ROS measurement.

Intracellular ROS was measured by a FACS-based method. Sorted neutrophils (5 × 10^5^ for each cell subset) or ER-Hoxb8 cells were incubated with 2.5 μg/mL (7 μM) dihydrorhodamine 123 (DHR123) (Thermo Fisher Scientific) in complete RPMI 1640 medium and stimulated by 50 nM Phorbol 12-Myristate 13-Actetate (PMA) (MilliporeSigma) for 20 minutes at 37°C. Cells were subsequently washed with PBS, and the fluorescence intensities of each subset/cells were measured by flow cytometry. Incubation (4°C) was used as a control.

Intracellular mitochondrial ROS generation in the form of superoxide was measured also by a FACS-based assay using MitoSOX red (Thermo Fisher Scientific). Sorted neutrophils (5 × 10^5^ for each cell subset) were incubated with 1 μM MitoSOX red in complete RPMI 1640 medium and were stimulated by 50 nM PMA (MilliporeSigma) for 10 minutes at 37°C. Cells were subsequently washed with PBS, and the fluorescence intensities of each subset/cells were measured by flow cytometry. Incubation (4°C) was used as a control.

Extracellular ROS measurement was performed by a fluorometric assay–based method using OxyBURST H2 HFF Green BSA (Thermo Fisher Scientific). Hoxb8 neutrophils (2 × 10^6^) or human neutrophil subsets (2 x 10^5^) were resuspended in KRP reaction buffer (phosphate-buffered saline, pH 7.4, with 1.0 mM Ca^2+^, 1.5 mM Mg^2+^, and 5.5 mM glucose; MilliporeSigma) in a 96-well plate. A total of 10 μL of OxyBURST H2 HFF Green BSA was added to each well for 2 minutes at 37°C and treated with 1 μM fMLP. Subsequently, release of extracellular ROS was measured by a continuous fluorescence increase excited at 488 nm and detected at 530 nm over a period of 120 minutes with 5-minute intervals on FLUOstar Omega microplate reader (BMG Labtech).

### Endothelial permeability.

Endothelial permeability assay was performed with In Vitro Vascular Permeability Assay (96-well format) from MilliporeSigma (catalog ECM644). Briefly, 100 μL of HUVECs in EGM-2 growth medium (Lonza, catalog CC-3162) at the concentration of 5 × 10^5^ cells/mL were seeded into collagen-precoated inserts for 24 hours in 5% CO_2_ tissue culture incubator at 37°C to reach a 100% confluence cell monolayer. HUVECs were primed with 10 ng/mL TNF-α for 4–6 hours before coincubating with 5000 sorted human neutrophil subsets or Hoxb8 neutrophils (1:10 cell-to-cell ratio of neutrophils to HUVECs) in the presence of 1 μM fMLP for 24 hours. Following the completion of permeability treatment, the inserts were transferred to a new receiver tray with 250 μL fresh EGM-2 media in each well and a FITC-dextran high–molecular weight dye of 75 μL at 1:40 dilution was applied to each insert. After incubating the plate at RT for 2 hours protected from light, the reaction was stopped by removing the inserts from the receive tray and 100 μL media (mixed with permeated FITC-dextran that crossed the monolayer) were transferred to wells of a black 96-well opaque plate for fluorescence measurement on FLUOstar Omega microplate reader (BMG Labtech). For effect of ROS inhibition on endothelial permeability, the assay was performed as described as mentioned above with addition of 25 μM of DPI, a general inhibitor of NOX2, to the Hoxb8 and HUVEC coculture.

### Phagocytosis.

Phagocytosis capacity of human neutrophil subsets and Hoxb8 neutrophils was measured by a FACS-based method using pHrodo red *E*. *coli* BioParticles conjugate (Thermo Fisher Scientific). Sorted neutrophils (5 × 10^5^ for each cell subset) or Hoxb8 neutrophils were incubated with 10 μL of the pHrodo beads (Thermo Fisher Scientific) in complete RPMI 1640 medium for 15 minutes in at 37°C. Cells were subsequently washed with PBS, and the fluorescence intensities of each subset/cells were measured by flow cytometry. Incubation (4°C) was used as a control.

### RNA-seq of Hoxb8 neutrophils.

Total RNA of 1 × 10^6^ Hoxb8 neutrophils of different maturation stages were extracted using an RNeasy Mini Kit (QIAGEN) according to the manufacturer’s instruction. RNA concentration was determined by NanoDrop spectrophotometer. Subsequently, poly-A selected mRNA libraries were sequenced on Illumina HiSeq4000 yielding 20 × 10^6^ to 30 × 10^6^ 150 b.p. paired end reads per sample. These were mapped to the mm10 genome using STAR with the options: “—runMode alignReads –outFilterMismatchNmax 2.” Uniquely mapped read pairs were counted over annotated genes using feature Counts with the options: “-T 18 -s 2 -Q 255.” Differential expression was then analyzed with DESeq2 (59). Variance stabilized (VST) counts for all DESeq2 differentially expressed genes, likelihood ratio test, and FDRs < 0.01 were used for dimensionality reduction and heatmaps. Gene set enrichment analysis was performed using 1-sided Fisher’s exact tests (as implemented in the ‘gsfisher’ R package; https://github.com/Tariq-K/RNA; commit ID: 040785745d2329c0ad46aeb315108070c6a90049). Hoxb8 raw RNA-seq was deposited at Gene Expression Omnibus (GEO) database with accession number GSE147910 (https://www.ncbi.nlm.nih.gov/geo/query/acc.cgi?acc=GSE147910).

### Detection of oxidized proteins of HUVEC with Hoxb8 coculture.

A total of 5 × 10^5^ HUVECs were seeded onto gelatin-precoated (MilliporeSigma, catalog G1393) 6-well tissue culture plates in 1.5 mL full EGM-2 medium (Lonza, catalog CC-3162) and cultured overnight in 5% CO_2_ tissue culture incubator at 37°C to reach a 100% confluence cell monolayer. Prior to the addition of Hoxb8 neutrophils, the cell monolayer was treated with 10 ng/mL human TNF-α (BioLegend) for 4–6 hours. Subsequently, HUVECs were washed twice with PBS and cocultured with 1 × 10^6^ Hoxb8 cells at D3 and D5 in EGM-2 medium and in the presence of 1 μM fMLP (MilliporeSigma, catalog F3506) with or without 25 μM of DPI for 2 hours. HUVECs, after removal of Hoxb8 cells and thorough wash with ice-cold PBS, were lysed in ice-cold RIPA buffer (50 mM Tris-HCl, 150 mM NaCl, 1% Nonidet P-40, 0.5% sodium deoxycholate [NaDoc], 0.1% sodium dodecyl sulfate [SDS], 1× cOmplete protease inhibitor cocktails [Roche], 1 μM Epoxomicin [Calbiochem], and 10 mM Nethylmaleimide) and centrifuged at 21,000*g* for 15 minutes to separate cell debris. Protein concentration was determined using Qubit Protein Assay Kit (Invitrogen). Oxidized protein was visualized by Western blotting using OxyBlot kit (MilliporeSigma, Chemicon) following the manufacturer’s instructions.

### Statistics.

Statistical analysis was performed using GraphPad Prism version 7.04 (GraphPad Software) as follows. Unpaired nonparametric Mann-Whitney *U* test was used to analyze 2 independent groups. Correlation of CD10^hi^ and CD10^lo^ LDNs before and after treatment was calculated by Spearman’s correlation coefficient. A nonparametric Wilcoxon test was used for statistical analysis to compare the difference of neutrophil populations between the baseline and follow-up studies. Nonparametric 1-way ANOVA Kruskal-Wallis test was used for statistical analysis in Figure 2D. Two-way ANOVA with Šidák’s multiple-comparisons was used for post hoc analysis. Data were presented with median ± IQR for neutrophil subset comparison. Otherwise, data were presented with mean ± SD. *P < 0.05, **P < 0.01, ***P < 0.001, and ****P < 0.0001.

Two-way MANOVA and Pillai’s trace statistic were performed to assess and correct sex and age influence of frequency difference of neutrophil subsets between GCA patients and HCs (Figure 2A and Supplemental Figure 3, A and B) using SPSS (SPSS version 24.0).

### Study approval.

The study was approved by local NHS hospital trust, where patients and HCs were recruited under the REC no. 15/SW/0313.

## Author contributions

LW performed all experiments, except as noted below; ZA set up Hoxb8 experiments; TK performed all computational analyses; KZ conducted IF microscopy; AH and SM collected clinical samples; HLE assisted with FACS analysis; DA and CM provided help with CyToF analysis; EBE generated human BM neutrophil data; EVG generated heatmap of selected genes in human neutrophils; IAU and RL devised and directed the study; and IAU, LW, and RL wrote the manuscript.

## Supplementary Material

supplemental data

## Version 1 09/22/2020

In-Press Preview

## Version 2 10/15/2020

Electronic publication
