# Supplementary material for: ROS-producing immature neutrophils in giant cell arteritis are linked to vascular pathologies
Source: JCI Insight. 2020 Oct 15;5(20):e139163. doi: 10.1172/jci.insight.139163 (PMC7605529; doi:10.1172/jci.insight.139163)
Supplement: supplemental data [file jciinsight-5-139163-s210.pdf]

Supplementary Materials:

Methods

Human bone marrow neutrophil isolation

Human bone marrow was obtained from rib fragments that were removed from lung cancer patients for surgical resection. This study was consented for tissue collection for research purposes at the Hospital of the University of Pennsylvania and The Philadelphia Veterans Affairs Medical Center after obtaining consents that had been approved by their respective Institutional Review Boards (60). Bone marrow neutrophils were isolated from bone marrow cell suspensions using positive selection of CD15+ or CD66b+ cells with microbeads according to the manufacturer's instructions (Miltenyi Biotec, Auburn, CA). Bone marrow cell suspension was obtained from the rib fragments that were removed from patients as part of their lung cancer surgery. The single cell suspension was obtained by vigorous pipetting of cells flushed from bone marrow and passing the disaggregated cells through a 70 μM nylon cell strainer. To exclude the possible contamination of common progenitors, neutrophils were isolated from a CD34-depleted population of bone marrow cells. Anti-CD15 antibody-conjugated magnetic microbeads (Miltenyi Biotec) were used for positive selection.

Heatmap generation of selected genes from published transcriptomic dataset

Selected genes from the Grassi. *et al* dataset (37) from three healthy human bone marrow donors and three healthy human venous blood donors were used to generate the heatmap with pheatmap R package (Supplementary Figure 6A).

Selected genes from enriched $CD10^+$ and $CD10^-$ LDN transcriptomes were analysed as the following. FastQ files were downloaded from the GEO database using accession no.

GSE139360. Adapter sequences were removed using trimgalore/0.4.4 (https://github.com/FelixKrueger/TrimGalore). Reads were mapped to the human genome GRCh38 version 91 using hisat2/2.1.0 (61) and quantified with featureCounts/1.6.3 from the Subread package (62). Gene expression values (Variance stabilised (VST) counts) were calculated using the vst function of DESeq2 (59). Heatmaps were generated with the pheatmap R package. (Supplementary Figure 6B&C)

Supplementary references:

60. Singhal S, Bhojnagarwala PS, O'Brien S, Moon EK, Garfall AL, Rao AS, et al. Origin and Role of a Subset of Tumor-Associated Neutrophils with Antigen-Presenting Cell Features in Early-Stage Human Lung Cancer. Cancer Cell. 2016;30(1):120-35.

61. Kim D, Langmead B, and Salzberg SL. HISAT: a fast spliced aligner with low memory requirements. Nat Methods. 2015;12(4):357-60.

62. Liao Y, Smyth GK, and Shi W. featureCounts: an efficient general purpose program for assigning sequence reads to genomic features. Bioinformatics. 2014;30(7):923-30.

Supplementary Figures

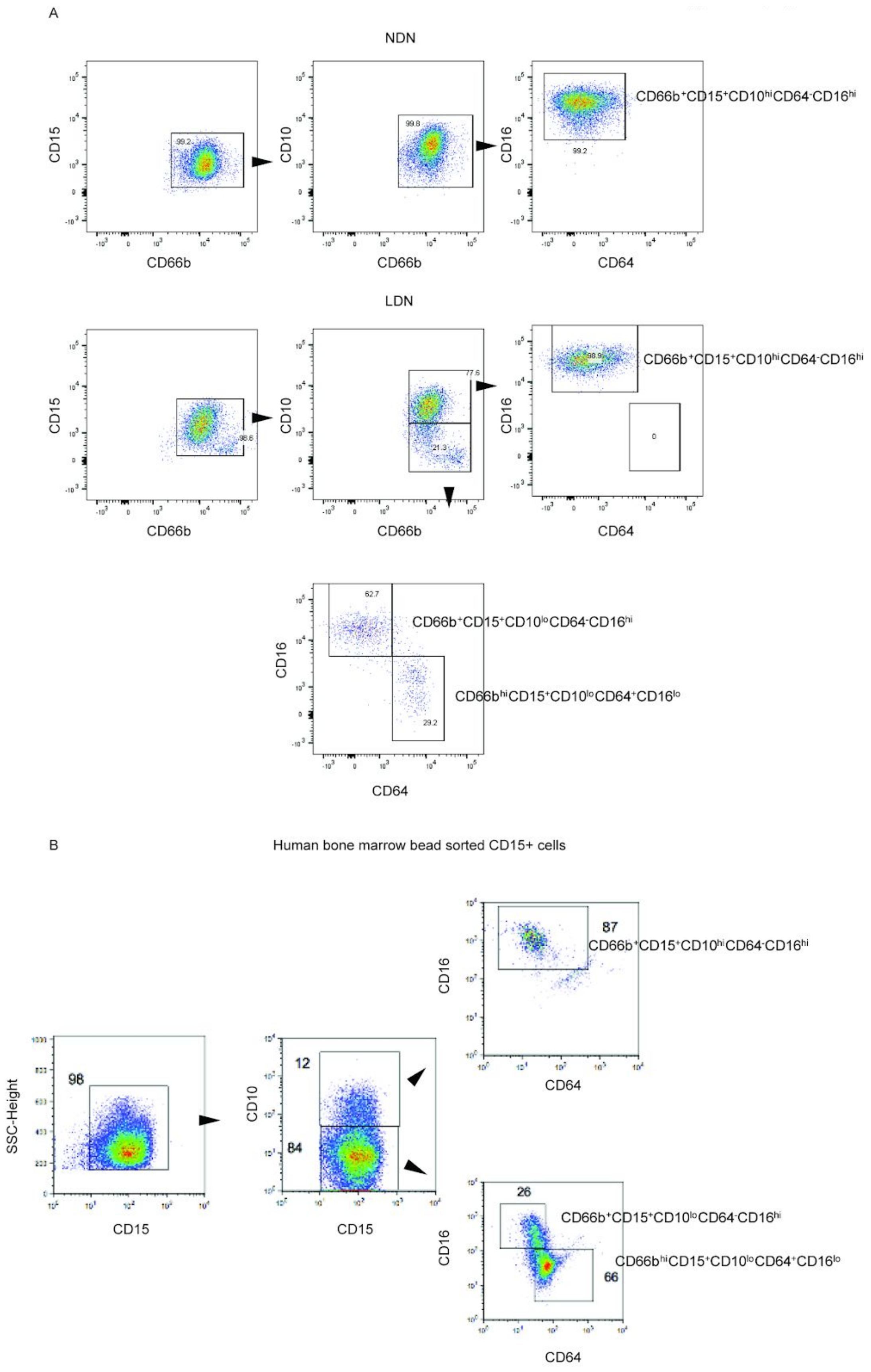

Figure 1 FACS gating strategy to isolate the four neutrophil populations A) in blood. B) Similar gating strategy was applied on bead sorted total neutrophils from human bone marrow to validate the use of CD64, CD10 and CD16 to define distinct neutrophil subsets.

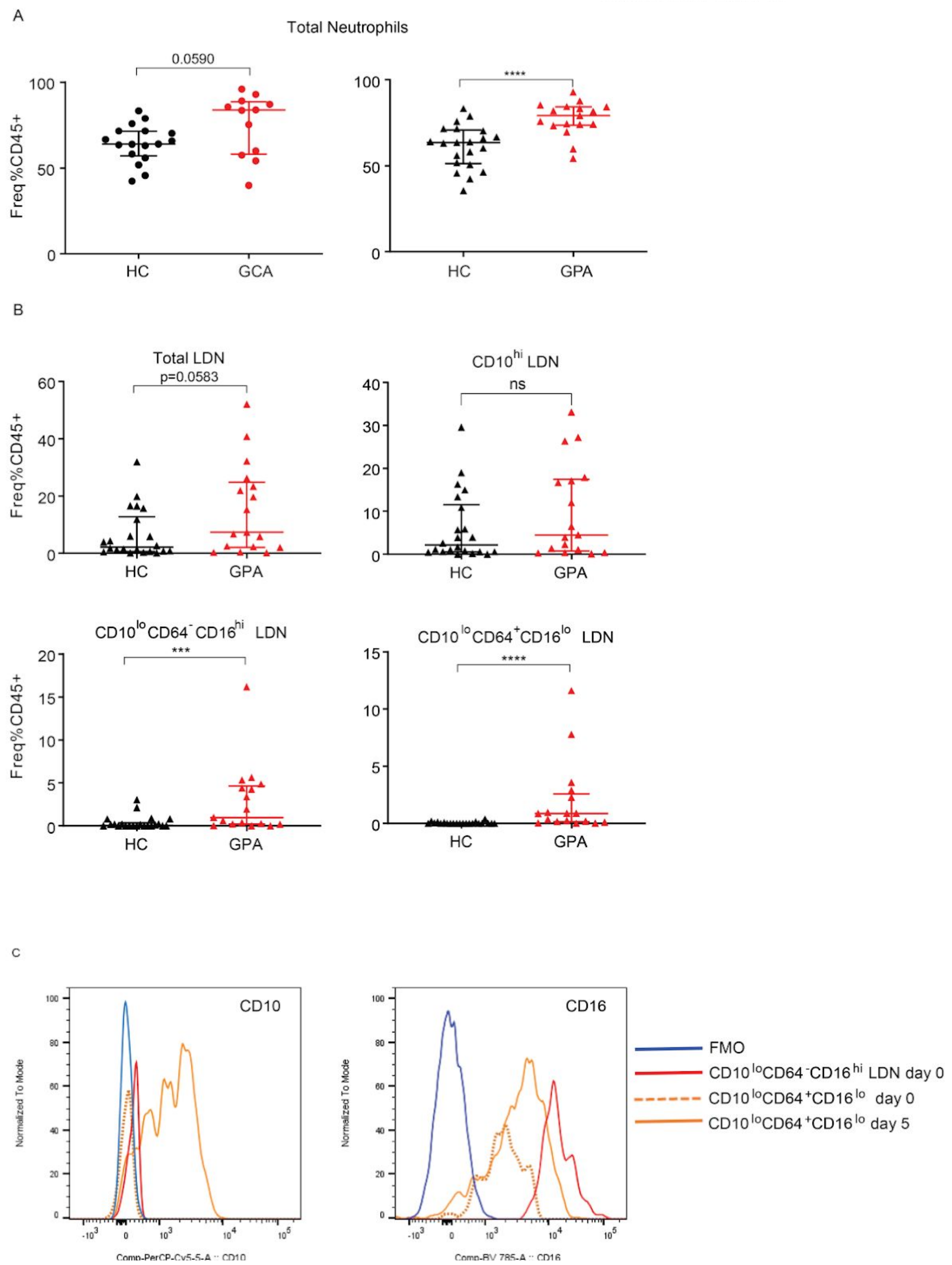

Figure 2 Total neutrophils in GCA and GPA and neutrophil subsets frequencies in GPA. A) Frequency of total neutrophils out of CD45+ immune cells in the blood between vasculitis patients and HCs. GCA n=13, HC=15 (for GCA); GPA n=17, HC=22 (for GPA). Data is presented with median± interquartile range. B) Neutrophil subset frequency comparison between GPA and HC. (GPA n=17 and HC n=22). Unpaired nonparametric Mann-Whitney test was used for statistical analysis. Data is presented with median± interquartile range. $^{*}p \leq 0.05$, $^{**}p \leq 0.01$, ***p

≤ 0.001, ****p ≤ 0.0001. C) $CD10^{lo}CD64^{+}CD16^{lo}$ expressed significant levels of CD10 and CD16 after 5 days *in vivo* culture in the presence of G-CSF. Solid blue line: The Fluorescence Minus One Control (FMO) for CD10 and CD16; red solid line: $CD10^{lo}CD64^{-}CD16^{hi}$ LDN at D0 in culture; orange dashed line: $CD10^{lo}CD64^{+}CD16^{lo}$ LDN at D0 in culture and orange solid line: $CD10^{lo}CD64^{+}CD16^{lo}$ LDN at D5 in culture. Expression of CD10 and CD16 is presented as FACS histograms. One representative from 3 independent experiments on FACS sorted neutrophil populations is shown.

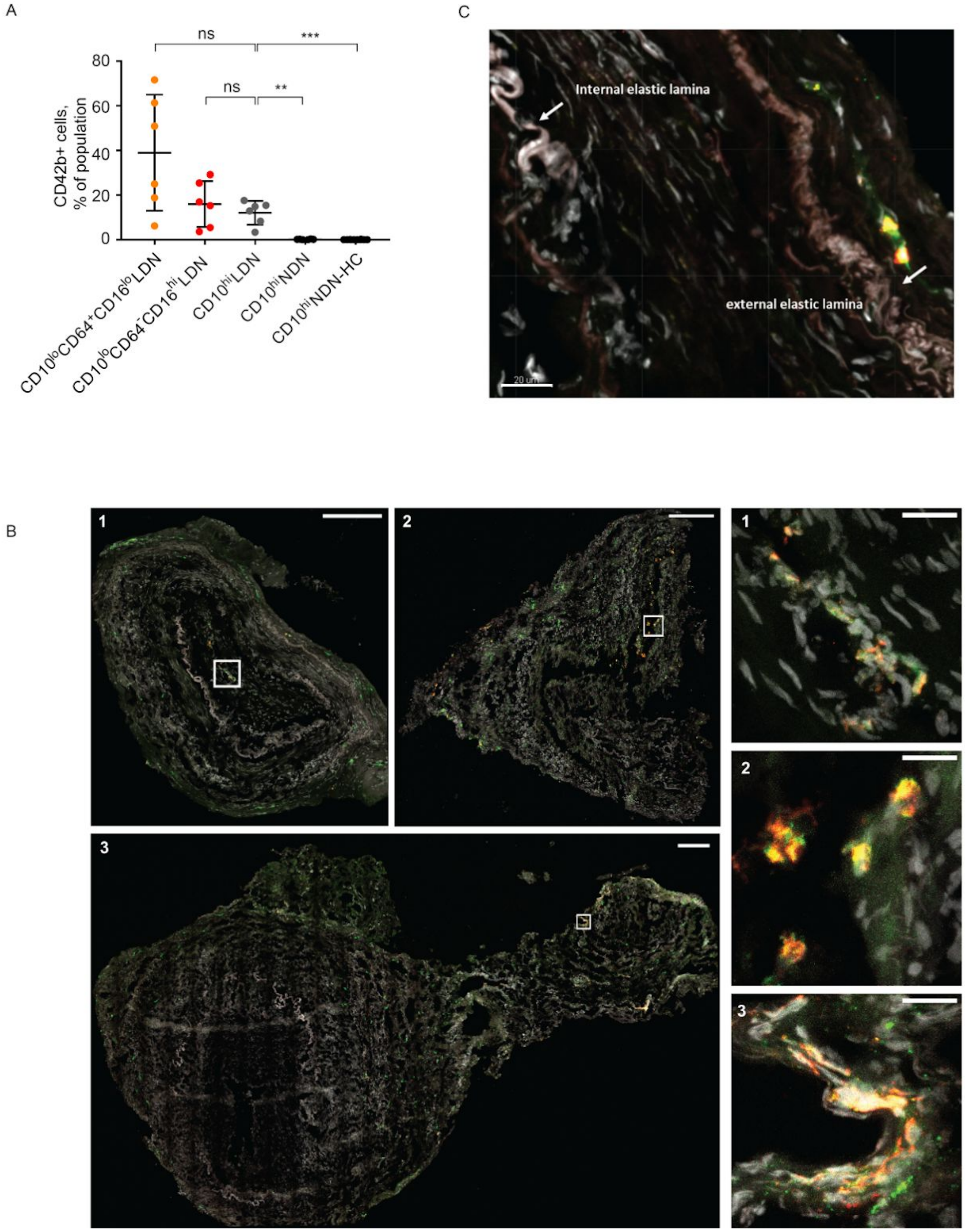

Figure 3 LDNs associate with platelets and confocal images of immature neutrophils distribution used for quantification. A) LDNs but not NDNs interacted with platelets. Data is presented with mean±SD. Nonparametric one way ANOVA Kruskal-Wallis test was used for statistical analysis. (GCA n=6, HC n=10). *P ≤0.05, **p ≤ 0.01, ***p ≤ 0.001, ****p ≤ 0.0001. B) Confocal images of temporal artery sections from 3 different GCA patients stained for neutrophil elastase (NE, red), CD15 (green) and Hoechst (grey) for DNA show the presence of

neutrophils within the lumen and tissue. These sections were used for quantification in Figure 3G. Zoomed in regions with neutrophils show mostly immature cells with unsegmented nucleus. Scale bar 20 µm. C) Immature neutrophils were found mainly in the lumen close to the internal elastica lamina and adventitia close to external lamina elastica but largely absent in the media. Breaks were observed in elastic lamina as indicated by the arrows.

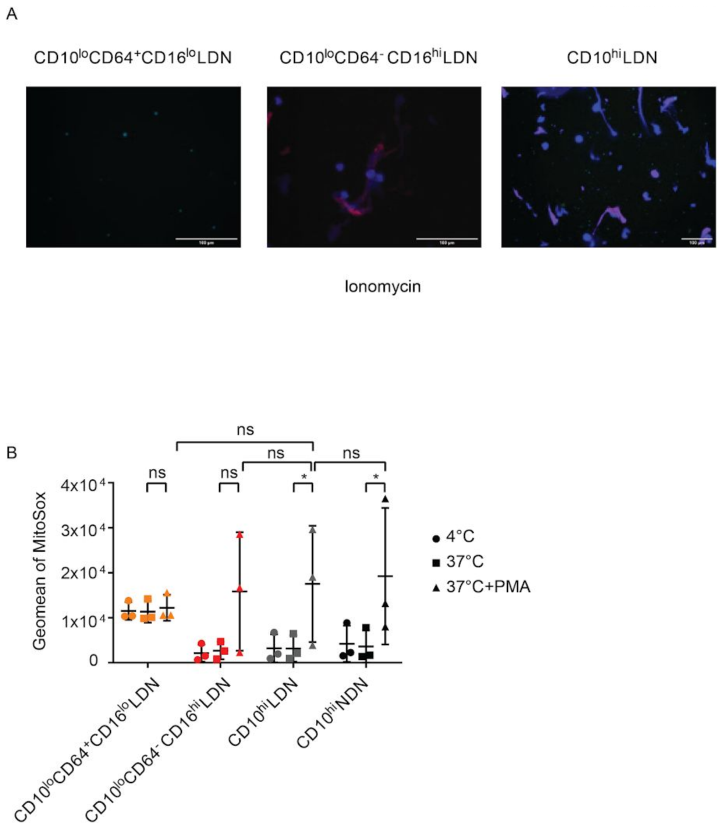

Figure 4 Confocal images of NET formation of LDN subsets and their mitochondrial ROS production. A) Merged images show NET formation of three FACS purified LDN populations. Red: Citrullinated Histone 3 (Cit-H3), Green: Elastase (NE) and Blue: DAPI. $CD10^{lo}CD64^{+}CD16^{lo}$ LDNs could not perform NETosis while the other neutrophil subsets were capable of NETosis. Representatives from at least three independent experiments are shown. B) Mitochondria-derived ROS was measured by MitoSOX™ Red. Immature LDNs were capable of generating mitochondrial ROS comparable to mature LDNs and NDNs. Data is presented with mean±SD .Three independent experiments were performed on FACS purified neutrophil populations from 3 GCA donors.

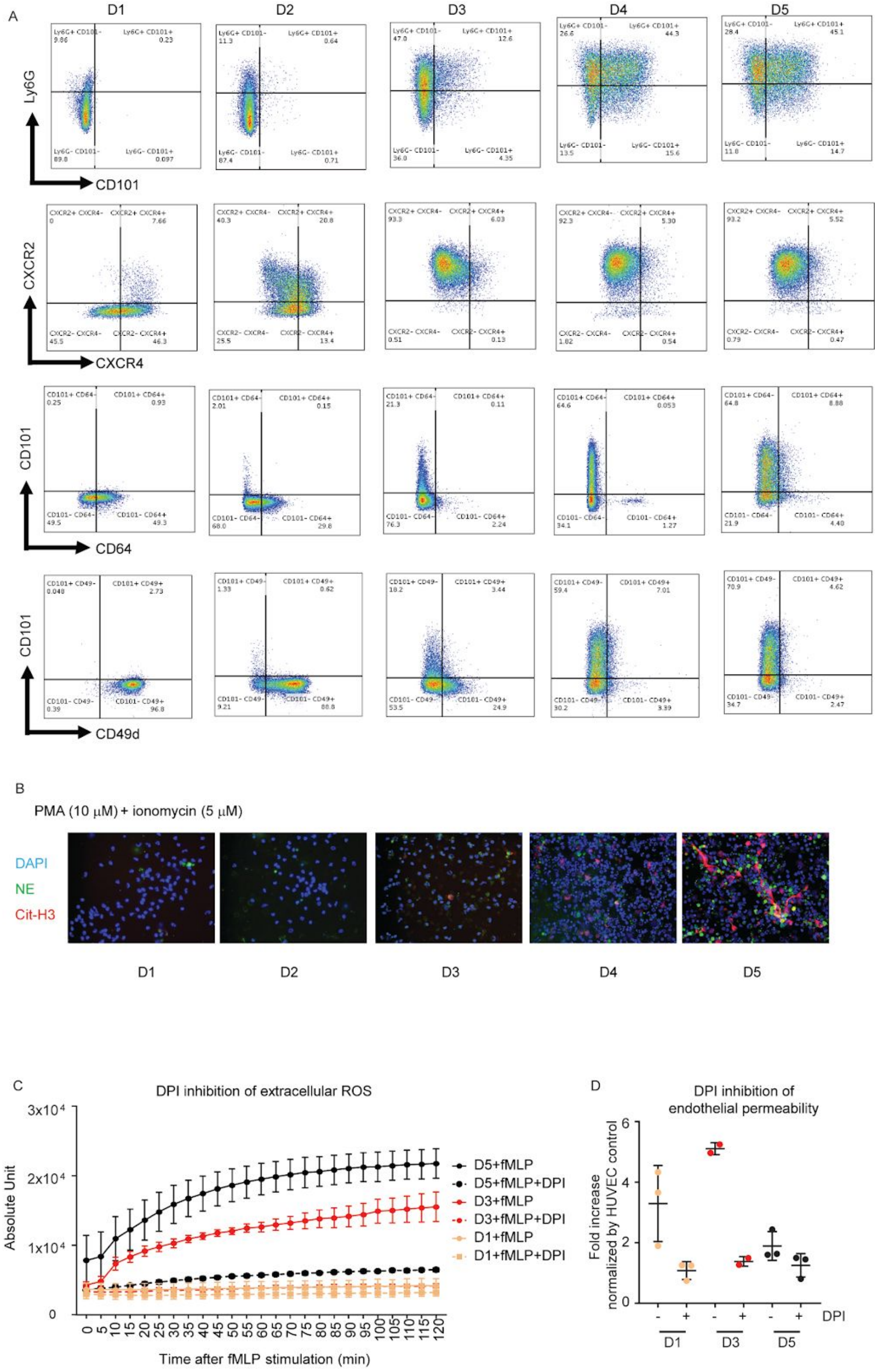
A
D1
D2
D3
D4
D5
Ly6G
CD101
CXCR2
CXCR4
CD101
CD64
CD101
CD49d
B
PMA (10 μM) + ionomycin (5 μM)
DAPI
NE
Cit-H3
D1
D2
D3
D4
D5
C
DPI inhibition of extracellular ROS
3x10^4
2x10^4
1x10^4
0
Absolute Unit
D5+fMLP
D5+fMLP+DPI
D3+fMLP
D3+fMLP+DPI
D1+fMLP
D1+fMLP+DPI
Time after fMLP stimulation (min)
D
DPI inhibition of endothelial permeability
Fold increase normalized by HUVEC control
DPI
D1
D3
D5

Figure 5 Validation of *ex vivo* Hoxb8 neutrophils as a useful system to study human neutrophils. A) Key surface markers in neutrophil maturation and development were conserved in the differentiation of ER-Hoxb8 neutrophils post G-CSF treatment from D1-D5. Scatter plots of one representative from at least three independent experiments were shown. B) Merged images show NET formation of Hoxb8 neutrophils from D1-D5 post differentiation. Red: Citrullinated Histone 3 (Cit-H3), Green: elastase (NE) and Blue: DAPI. Mature D5 Hoxb8 neutrophils were able to induce NET potently upon stimulation. Representatives from at least three independent experiments were shown. C) Addition of 25 μM DPI inhibited the generation of extracellular ROS in ER-Hoxb8 neutrophils. D) Extracellular ROS production at 120 minutes in the presence of 25 μM DPI was compared across ER-Hoxb8 neutrophils of different days of maturation. Data is presented with mean±SD

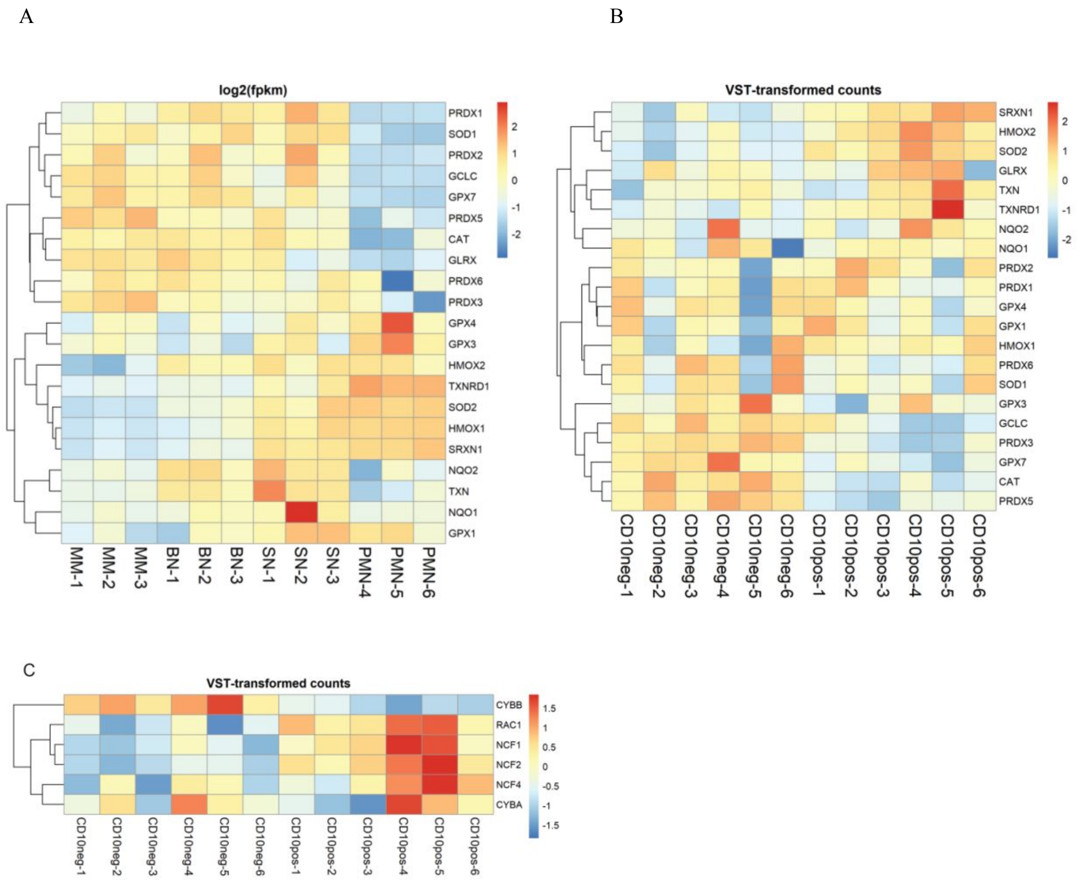

Figure 6 Expression comparison of selected anti-oxidant and NOX2 complex genes with published human neutrophil dataset. A) RNA expression in log2(FPKM) of selected genes from the Grassi *et al*. dataset, centred and scaled by genes, representing three healthy human bone marrow donors (donor 1-3) and three healthy human venous blood donors (donor 4-6) (37). MM = metamyelocytes, BN = band neutrophil, SN = segmented neutrophil, PMN = blood neutrophils. B) RNA expression in VST-transformed count values of selected anti-oxidant genes and C) NOX2 complex from the Mistry *et al*. dataset, centered and scaled by genes, representing CD10neg and CD10pos low-density granulocytes from six SLE patients (38).

Supplementary tables

Table 1 CyToF antibody panel

Metal	Antibody	Clone	Chosen tag	Company	Cat. Number
89	CD45	HI30	Y	Fludigm	3089003B
141	CD42b	HID1	Pr	BioLegend	303902
141	CD235ab	DREG-56	Pr	Fluidigm	3141001B
142	CD19	HIB19	Nd	Fluidigm	3142001B
143	HLADR	L243	Nd	Fluidigm	3143013B
144	CD35	E11	Nd	BioLegend	333402
145	CD31	WM59	Nd	Fluidigm	3145004B
146	CD64	10.1	Nd	Fluidigm	3146006B
147	CD54	HCD54	Sm	BioLegend	322702
148	CD16	3G8	Nd	Fluidigm	3148004B
149	CD66a	CD66a-B1.1	Sm	Fluidigm	3149008B
151	CD123	6H6	Eu	Fluidigm	3151001B
152	CD66b	80H3	Sm	Fluidigm	3152011B
153	CD62L	DREG-56	Eu	Fluidigm	3153004B
154	CD163	GH1/61	Sm	Fluidigm	3154007B
156	CD86	IT2.2	Gd	Fluidigm	3156008B
158	CD10	HI10a	Gd	Fluidigm	312202
159	CD11c	Bu15	Tb	Fluidigm	3159001B
160	CD14	M5E2	Gd	Fluidigm	3160006B
161	CD80	2D10.4	Dy	Fluidigm	3161023B
162	CD88	S5/1	Dy	BioLegend	344302
163	CD181	5A12	Dy	BD Biosciences	555937
164	Siglec-8	7C9	Dy	BioLegend	347102
165	CD101	BB27	Ho	Fluidigm	3165029B

166	CD182	eBio5E8-C7-F10	Ho	eBioscience	14-1829-85
168	CD206	15.2	Er	Fluidigm	3168008B
169	CD33	WM53	Tm	Fluidigm	3169010B
170	CD3	UCHT1	Er	Fluidigm	3170007B
172	CD15	W6D3	Yb	Fluidigm	3172021B
173	CD184	12G5	Yb	Fluidigm	3173001B
174	CD49d	9F10	Yb	Fluidigm	3174018B
175	CD274	29E.2A3	Lu	Fluidigm	3175017B
176	CD56	NCAM16.2	Yb	Fluidigm	3176008B
209	CD11b	ICRF44	Bi	Fluidigm	3209003B

Table 2A Clinical features of diagnosis (n=8) and flare (n=6)

	GCA	HC
Demographics		
Age (ys Mean± S.E.M)	70.9±2.6	62.9±1.9
Gender	8M 6F	3M 13F
Smoking history		
Ex-smoker	33%	-
current smoker	1.6%	-
never smoked	50%	-
Cardiovascular		
hypertension	50%	-
Symptoms		
scalp tenderness	94%	-

headaches	94%	-
morning stiffness (up to 60mins)	26%	-
Ultrasound of temporal arteries		
Halo on both sides	38%	-
Halo on single side	55%	-
BVAS (Mean± S.E.M)	6.74±0.9	-
		-
VDI (Mean± S.E.M)	2.9±0.78	
Haematology and biochemistry (Mean± S.E.M)		
Haemoglobin (g/L)	131±4.9	-
Neutrophil (x10^9 L)	10±2.75	-
Platelets (x10^9 L)	346.6±39.7	-
CRP (g/L)	29.1±17.7	-

Table 2B Clinical features of diagnosis (n=12) and flare (n=5)

	GPA	HC
Demographics		
Age (ys Mean± S.E.M)	57.3±3.9	54±3.4
Gender	7M 10F	6M 16F
Smoking history		
Ex-smoker	33%	-
current smoker	6%	-
never smoked	60%	-

Cardiovascular		
hypertension	26%	-
Symptoms		
Nasal crusting	60%	-
Epistaxis	53%	-
Paranasal sinus involvement	53%	-
history of sinusitis	40%	-
cANCA+ve PR3+ve	60%	-
pANCA+ve MPO+ve	6%	-
pANCA+ve MPO-ve	6%	-
BVAS (Mean± S.E.M)	13.4±1.3	-
VDI (Mean± S.E.M)	0.76±0.36	-
Haematology and biochemistry (Mean± S.E.M)		
Haemoglobin (g/L)	122±4.9	-
Neutrophil (x10^9 L)	8.27±0.75	-
Platelets (x10^9 L)	342.8±29	-
CRP (g/L)	53.7±14.9	-
Creatinine (g/L)	93.1±16.8	-

Table 3A Two way multivariate analysis of variance (MANOVA) on age and gender on neutrophil subset frequency difference

Multivariate Tests[a]							
Effect		Value	F	Hypothesis df	Error df	Sig.	Partial Eta Squared
Intercept	Pillai's Trace	0.122	.870[b]	4.000	25.000	0.496	0.122
Condition	Pillai's Trace	0.322	2.969[b]	4.000	25.000	0.039	0.322
Gender	Pillai's Trace	0.139	1.009[b]	4.000	25.000	0.422	0.139
Age	Pillai's Trace	0.091	.628[b]	4.000	25.000	0.647	0.091

a. Design: Intercept + Condition + Gender + Age

b. Exact statistic

Table 3B Corrected two way MANOVA analysis of each neutrophil subset on age and gender

Corrected Tests of Between-Subjects Effects							
Source	Dependent Variable	Type III Sum of Squares	df	Mean Square	F	Sig.	Partial Eta Squared
Corrected Model	$CD10^{hi}$	1820.965[a]	3	606.988	3.386	0.032	0.266
	TotalLDN	6871.363[c]	3	2290.454	4.473	0.011	0.324
	$CD10^{lo}CD64^{-}$	532.976[d]	3	177.659	2.098	0.123	0.183
	$CD10^{lo}CD64^{+}CD16^{l}$	66.797[e]	3	22.266	2.949	0.049	0.240
Intercept	$CD10^{hi}$	400.764	1	400.764	2.235	0.146	0.074
	TotalLDN	155.959	1	155.959	0.305	0.585	0.011
	$CD10^{lo}CD64^{-}$	22.120	1	22.120	0.261	0.613	0.009
	$CD10^{lo}CD64^{+}CD16^{l}$	0.250	1	0.250	0.033	0.857	0.001
Condition	$CD10^{hi}$	1805.340	1	1805.340	10.069	0.004	0.265
	TotalLDN	5994.366	1	5994.366	11.705	0.002	0.295
	$CD10^{lo}CD64^{-}$	320.048	1	320.048	3.779	0.062	0.119
	$CD10^{lo}CD64^{+}CD16^{l}$	35.702	1	35.702	4.729	0.038	0.144
Gender	$CD10^{hi}$	158.847	1	158.847	0.886	0.355	0.031
	TotalLDN	693.618	1	693.618	1.354	0.254	0.046
	$CD10^{lo}CD64^{-}$	35.431	1	35.431	0.418	0.523	0.015
	$CD10^{lo}CD64^{+}CD16^{l}$	4.172	1	4.172	0.553	0.463	0.019
Age	$CD10^{hi}$	234.131	1	234.131	1.306	0.263	0.045
	TotalLDN	39.882	1	39.882	0.078	0.782	0.003
	$CD10^{lo}CD64^{-}$	30.687	1	30.687	0.362	0.552	0.013
	$CD10^{lo}CD64^{+}CD16^{l}$	0.186	1	0.186	0.025	0.876	0.001
Error	$CD10^{hi}$	5020.071	28	179.288			
	TotalLDN	14338.868	28	512.102			
	$CD10^{lo}CD64^{-}$	2371.587	28	84.700			
	$CD10^{lo}CD64^{+}CD16^{l}$	211.399	28	7.550			
Total	CD10hi	11777.638	32				
	Total LDN	34063.486	32				
	$CD10^{lo}CD64^{-}$	3471.397	32				
	$CD10^{lo}CD64^{+}CD16^{l}$	345.227	32				
Corrected Total	$CD10^{hi}$	6841.036	31				
	Total LDN	21210.231	31				
	$CD10^{lo}CD64^{-}$	2904.562	31				
	$CD10^{lo}CD64^{+}CD16^{l}$	278.196	31				

a. R Squared = .266 (Adjusted R Squared = .188)

b. R Squared = .269 (Adjusted R Squared = .191)

c. R Squared = .324 (Adjusted R Squared = .252)

d. R Squared = .183 (Adjusted R Squared = .096)

e. R Squared = .240 (Adjusted R Squared = .159)

Table 4 FACS antibody panel

Conjugation	Antibody	Clone	Company	Cat. Number
Alex-Flour® 700	CD3	OKT3	Biolegend	317339
APC/Cy7	CD3	OCHT1	Biolegend	300425
PerCP Cy5.5	CD10	HI10a	Biolegend	312216
BV510	CD11b	ICRF44	Biolegend	301334
FITC	CD14	10.1	Biolegend	305005
BV650	CD14	M5F2	Biolegend	301835
APC/Cy7	CD14	M5F2	Biolegend	301819
FITC	CD15	W6D3	BD Pharmingen	562370
PE/Dazzle 594	CD15	W6D3	Biolegend	323038
BV785	CD16	3G8	Biolegend	302045
PeCy7	CD45	IV N816	BD Pharmingen	557748
BV650	CD45	HI30	Biolegend	304001
PE Dazzle	CD49d	9F10	Biolegend	304326
BV605	CD62L	DREG-56	Biolegend	304834
PE	CD64	10.1	Biolegend	305007
BV421	CD66b	G10F5	BD Biosciences	562940
PE/Cy7	CD101	BB27	Biolegend	331013
APC	CD114	LMM741	Biolegend	346107
FITC	CD116	4H1	Biolegend	305906
PE	CXCR4	12G5	Biolegend	306505
APC	Siglec8	7C9	Biolegend	347105
PE	Siglec8	7C9	Biolegend	347111
APC	Ki67	20Raj1	ThermoScientific	17-5699-4 2
BV 711	Ly6G	1A8	Biolegend	127643

PE-Cyanine7	CD101	Moushi101	Life Technologies	25-1011-8<br>2
PE	CXCR2	SA044G4	Biolegend	149303
BV 421	CXCR4	L276F12	Biolegend	146511
Alexa Fluor® 647	CD64	X54-5/7.1	Biolegend	139321
APC	CD49d	R1-2	Biolegend	103621
BV 785	Ly-6C	HK1.4	BioLegend	128041
